# Supplementary material for: Spatio-temporal dynamics of Hendra virus in Australia reveal stable maintenance of diverse viral clades among Pteropus bats
Source: Nat Microbiol. 2026 Apr 7;11(4):851–66. doi: 10.1038/s41564-025-02254-7 (PMC13056563; doi:10.1038/s41564-025-02254-7)
Supplement: Supplementary file 1 — Supplementary Results, Discussion, Tables 1–8 and Figs. 1–5. [file 41564_2025_2254_MOESM1_ESM.pdf]

nature microbiology

Supplementary information

https://doi.org/10.1038/s41564-025-02254-7

Spatio-temporal dynamics of Hendra virus in Australia reveal stable maintenance of diverse viral clades among *Pteropus* bats

In the format provided by the authors and unedited

Supporting Information for

Spatio-temporal dynamics of Hendra virus in Australia reveals stable maintenance of diverse viral clades among *Pteropus* bats

Authors list

Claude Kwe Yinda[1#], John-Sebastian Eden[2,3#], Erica Prates[4#], Anna Vlot[4], Sarah van Tol[1], Sarah L. Anzick[5], Jianning Wang[6], Kim Halpin[6], Benny Borremans[7], Tamika J. Lunn[8,9], Kent Barbian[5], Brown Bulloch[1], Benjamin Greene[1], Kimberly Meade-White[1], Trenton Bushmaker[1], Caylee A. Falvo[10], Daniel E. Crowley[10], Devin N. Jones-Slobodian[11], Manesh Shah[12], Mirko Pavicic[4], William Carr[13], Craig Martens[5], Daniel Jacobson[4], Bat One Health group, Raina K. Plowright[10], Alison J. Peel[3,14,15$], Vincent J. Munster[1$] *

Affiliations

[1]*Laboratory of Virology, Division of Intramural Research, National Institutes of Health, Hamilton, MT, USA*

[2]*Centre for Virus Research, Westmead Institute for Medical Research, Westmead, NSW, Australia.*

[3]*Sydney Institute for Infectious Diseases, The University of Sydney, Sydney, NSW, Australia.*

[4]*Oak Ridge National Laboratory, Oak Ridge, TN, USA*

[5]*Genomics Research Section, Research Technologies Branch, Division of Intramural Research, National Institute of Allergy and Infectious Diseases, National Institutes of Health, Hamilton, MT, USA*

[6]*Australian Centre for Disease Preparedness, Commonwealth Scientific and Industrial Research Organisation (CSIRO), Canberra, Australia*

[7]*Wildlife Health Ecology Research Organization, San Diego, USA.*

[8]*Odum School of Ecology, University of Georgia, Athens GA, USA*

[9]*Center for the Ecology of Infectious Diseases, University of Georgia, Athens GA, USA*

[10]*Department of Public and Ecosystem Health, College of Veterinary Medicine, Cornell University, Ithaca NY, USA*

[11]*Department of Ecology, Montana State University, Bozeman, MT, USA*

[12]*Biochemistry & Cellular And Molecular Biology, The University of Tennessee, Knoxville TN, USA*

[13]*Department of Biology, Medgar Evers College, City University of New York, New York NY, USA*

[14]*Centre for Planetary Health and Food Security, Griffith University, Nathan, Queensland, Australia*

[15]*Sydney School of Veterinary Science, University of Sydney, Sydney, NSW, Australia*

[#] These authors contributed equally

[$] These authors jointly supervised this work

* Corresponding author: Vincent Munster

Rocky Mountain Laboratories, NIAID, NIH

903 S 4th St, Hamilton, MT 59840

Email: vincent.munster@nih.gov

1. Supporting Results

We tested the temporal structure in the presence of different clades using a permutation test to address two specific questions: (1) How unlikely is it that clade B is not observed before 2018 if it was in fact present throughout the sampling period, and (2) Do the relative proportions of clades change over time? The results showed that the proportion of permutations for which clade B is not observed before 2018 was 0.022, which means that it is statistically unlikely that clade B was present before 2018.

Supp Fig. 8 show the permuted and observed proportions of each clade out of the four clades within each 6-month period (excluding years before 2017 due to low sample sizes. There is no statistical support for the existence of periods during which the proportion of a clade is different from that expected given a constant distribution of clades over time (with the exception of clade B samples in Jul-Dec 2017).

2. Supporting Discussion

Here, we discuss the potential functional impact of amino acid substitutions identified in the HeV genomic data, focusing on the 16 lineage-defining (LD) substitutions. These variants were prioritized based on recurrency and/or their location in known/predicted functional regions, indicating relevance to viral replication, host interaction, or immune evasion. Using structural analysis and priori literature, we propose hypotheses regarding their possible roles in altering protein structure and dynamics, post-translational modifications, oligomerization, and protein-protein interactions with host or viral components. We also provide, as Supplementary Data, the files with all the substitutions mapped to HeV strains and their corresponding clades.

2.1. Nucleoprotein (N)

The HeV nucleoprotein (N) is a critical structural and functional component of the viral replication machinery. As the first protein encoded in the HeV genome, N encapsidates the viral RNA to form a protective nucleocapsid, which serves as the essential template for transcription and replication by the viral RNA-dependent RNA polymerase complex. Structurally, the HeV N protein adopts a conserved paramyxoviral fold comprising N-terminal and C-terminal globular domains connected by flexible arms

and flanked by an intrinsically disordered C-terminal tail. These features enable the N protein to mediate both RNA binding and dynamic multimerization, forming helical or ring-shaped assemblies that fully enclose the viral genome. The recent 3.5 Å cryoEM structure of RNA-bound HeV N revealed a previously uncharacterized double-ring assembly and helps to evaluate potential effects of substitutions identified in our genomic dataset [1].

Noticeably, while we do not find LD substitutions in N, the four substitutions relative to the reference genome are persistent in the population, and all of them result in the amino acids that are present in HeV-g2 and/or NiV genomes. We discuss potential functional importance below:

- **D6E:** This substitution is part of a negatively charged patch (Asp3, Asp6, and Glu7) in the Nt-arm that lines the central cavity of the double-ring assembly. The substitution is chemically conservative and likely has a minor impact. Interestingly a glutamate is found in HeV-g2 and NiV sequences.
- **E498G**, **R512G**, and **P518S**: These substitutions are located in intrinsically disordered C-terminus of N, outside the core α-MoRE region (residues 473–490) that directly binds the phosphoprotein's P_{XD} domain [2]. While these sites are likely not essential for primary P–N interaction, they may influence the flexibility, electrostatics, and secondary binding potential of N-tail. The E498G and R512G mutations add conformational flexibility and remove charged side chains, potentially affecting transient electrostatic interactions with the phosphoprotein or other partners. The P518S substitution replaces a structurally rigid proline with a more flexible serine, possibly increasing local disorder. Collectively, these mutations could subtly alter the conformational ensemble of N-tail, potentially affecting regulatory functions such as phase separation, replication dynamics, or interactions with host factors, without disrupting the core P_{XD}–N interface. Noticeably, R512G and P518S result in the amino acid present in the HeV-g2 genome.

2.2. Phosphoprotein (P) and accessory V and W proteins

The phosphoprotein (P) is a key component of the HeV replication complex, serving as a cofactor for the viral RNA-dependent RNA polymerase (L). In addition, it is a chaperone for the nucleoprotein (N), binding to its monomeric form and preventing self-assembly. The V and W accessory proteins are generated through co-transcriptional editing of the *P* gene mRNA, where the viral polymerase stutters at a conserved editing site, inserting one or two non-templated guanine residues, respectively [3]. This mechanism raises the possibility of epistasis between the *L* and *P* genes and the co-occurrence of the LD mutations in L and P/V/W proteins observed in our dataset, which motivates future investigation.

Due to RNA editing-induced frameshifts, the P, V, and W proteins have the same N-terminal domain (NTD) but unique C-terminal domains (CTD). The shared NTD includes a highly conserved region between HeV and NiV P (aa. 81-120) essential for its polymerase cofactor activity. Consistent with this functional importance, we did not observe amino acid substitutions in this region.

A major strategy of immune evasion in henipaviruses is the suppression of type I interferon (IFN-I) responses and downstream interferon-stimulated gene (ISG) expression. These functions are primarily mediated by the V and W proteins, though the N protein and matrix protein (M) also contribute to immune modulation [4]. For example, adopting a zinc finger conformation, V-CTD binds MDA5, an IFN-inducible RNA helicase, thereby blocking IFN-β induction [5]. Similarly, the V protein binds to both the CARD domains of RIG-I and the SPRY domain of TRIM25, an E3 ubiquitin ligase essential for RIG-I activation, reducing IFN-β signaling independently of other pathways [6,7]. Furthermore, V-CTD blocks nuclear translocation of IRF3, reducing NF-κB–driven transcription [5,8].

The shared NTD of P/V/W is largely intrinsically disordered (IDR), enabling structural flexibility and versatile interactions with diverse host proteins. Structural data show that the V protein undergoes a disorder-to-order transition, acquiring α-helical structure and compactness upon binding host nuclear transport proteins such as importin α2/β1 and exportin-1/Ran-GTP [9]. In HeV, V-NTD directly binds

STAT1 and STAT2, blocking their nuclear import [10]. In NiV these transcription factors are instead sequestered into cytoplasmic inclusion bodies [11]. Interference with STAT1- and STAT2-mediated IFN-α/γ signaling is a conserved strategy across paramyxoviruses.

We identified five LD mutations in the shared IDR of P/V/W proteins and discuss their functional implications:

- **R149C:** Located within the STAT1-binding motif (aa 101–160) in the IDR, this substitution specific to Clade A may influence the efficiency of STAT1 antagonism [12]. Notably, a cysteine is present at the corresponding site in HeV-g2 and NiV. *In vitro* studies have shown that HeV V does not bind recombinant STAT1, suggesting that this interaction may depend on additional host factors or post-translational modifications [9]. Consistent with this, we were unable to generate a high-confidence structural model for the V–STAT1 complex.
- **G219E:** This site is located within a predicted phase transition region (aa. 200–310), termed PNT3, implicated in forming amyloid-like fibrils *in vitro* and in cellular context [13]. It has been proposed that fibrils may act as molecular traps, sequestering host proteins involved in antiviral immunity, such as those in the interferon pathway. Site 219 is close to a triple tyrosine motif (EYYY, aa. 210-213) identified as the key amyloidogenic region. Interestingly, NiV does not form hydrogels and encodes a glutamate at this position, implying a potentially conserved functional role across henipaviruses. This substitution is Clade C-defining and persists in Clade D.
- **D304G and K317E:** These substitutions represent a chemically significant change within the IDR of P/V/W. Site 304 is also part of PNT3 and the D304G substitution results in a residue found at the same position in NiV. D304G is a Clade A-defining substitution and K317E is a Clade C-defining substitution, also predominant in Clade D.
- **P352S:** Although proline and serine are abundant amino acids in IDRs, they exert very different effects in conformational flexibility and compactness. Proline introduces rigidity, while serine

allows greater structural adaptability and can be phosphorylated. The P352S substitution may thus alter local structure or introduce a post-translational modification site. Indeed, a prediction using NetPhos-3.1[14] is positive for phosphorylation in the serine at that site (score 0.992). Moreover, this residue lies adjacent to a region with intermediate pLDDT scores [15] in AlphaFold predictions [16] (Suppl. Fig. 11), potentially suggesting a transient α-helical propensity, a hallmark of many IDR-mediated interaction motifs. The adjacent sequence is highly conserved between HeV and NiV (87% identity in the 10 flanking residues; Supp. Fig. 9). Notably, this site is also near PNT3 (aa. 200–310) [13]. P352S is prevalent in Clade B strains, being present in strain 84, used in in vitro assays (ARTOW006_VTM_U_14_1).

In addition to lineage-defining (LD) variants, we identified other substitutions in the P/V/W proteins that may have functional relevance:

- **H139Y**: This substitution occurs within the intrinsically disordered STAT1-binding motif (aa. 101–160) and may influence the efficiency of STAT1 antagonism [12]. This substitution is present in Clade D, strains ARTOW004_AVL_U_3_1, ARTOW004_AVL_U_4_1, HORSE002.
- **R170G:** This residue is positioned near a region known to undergo disorder-to-order transition upon binding to exportin-1 (aa. 174-192) [9], a host nuclear export factor. R170G is prevalent across all HeV clades in our dataset, suggesting possible selective retention or functional adaptation.

2.3. Matrix (M) protein

The matrix (M) protein is considered the primary driver of viral morphogenesis and lines the inner leaflet of the viral membrane, binding to the ribonucleoprotein complex and the cytoplasmic tail of the glycoprotein G. Beyond its scaffolding role, HeV M engages host proteins, including fibrillarin [17] and ANP32B [18], and undergoes regulated nuclear–cytoplasmic trafficking via defined NLS and NES signals, a process essential for virion assembly [19].

In our dataset, M is highly conserved. The only recurrent observed substitution is N31D, with the aspartate present in all sequences except the human reference sequence, KY425627.

- **N31D:** This site lies within the N-terminal region, mapping to the convex surface of M, a site potentially involved in protein-protein interactions, such as binding to the ribonucleoprotein complex. The substitution converges with the corresponding residue in NiV and HeV-g2, introducing a negative charge that could alter such interactions. The region spanning residues 1-44 is flexible and is not captured in the recently X-ray-solved structure of M (PDB id: 6BK6) [19].

2.4. Fusion (F) protein

The fusion (F) protein is essential for initiating infection, syncytia formation, and is a major target for host immune responses and antiviral strategies [20]. Synthesized as an inactive precursor (F_0), it is cleaved by host proteases into the F_1 and F_2 subunits, which remain linked by disulfide bonds. Upon receptor binding to the attachment G protein, F-trimer undergoes a series of large conformational changes that drive the merger of the viral envelope with the host cell membrane, enabling delivery of the viral ribonucleoprotein complex into the cytoplasm.

We identify two substitutions in F in our genome data, namely:

- **S88L:** Located at the surface of apex F2 in the prefusion state, the substitution changes the hydropathy at that site (PDB id: 8DNR) [21]. Although there is no direct evidence of its functional implications, the chemical change could influence the fusion process and protein-protein interactions, including with G or with neutralizing antibodies, if it is part or adjacent to an epitope. Indeed, in NiV F, the threonine at that site forms a hydrophobic contact with the 1A9 antibody (PDB id: 7UPK) [22]. The substitution is present in Clade C, strains ACTOW001_AVL_U_26_1, RSIM001_NB_U_25, and ARLIS002_AVL_U_36_1.

- **A141T:** This residue lies within the heptad repeat A (HRA) region of F2, a critical element involved in membrane fusion [23]. It is buried and participates in hydrophobic interactions that stabilize the HRA attachment to the DIII domain in the prefusion state (PDB id: 8DNR) [21]. The substitution could disrupt these interactions and thereby influence fusion efficiency. The substitution is present in Clade D, strains ARTOW004_AVL_U_38_1, ARTOW004_AVL_U_3_1, and ARTOW004_AVL_U_4_1.

Notably, both substitutions, S88L and A141T, converge on the same residues present in the Cedar virus (CeV) F protein.

2.5. Attachment glycoprotein (G)

The HeV G glycoprotein mediates virion attachment to host cells to establish infection. It functions as a tetramer, organized as a homodimer of dimers on the viral surface. Structurally, G comprises a stalk region that anchors the protein to the viral membrane and a globular head domain that binds to the host receptor, ephrin-B2 or ephrin-B3. Interaction with the host receptor initiates conformational changes that allow for interactions with the fusion protein (F) and its activation, triggering membrane fusion and viral entry [20].

We identify two LD mutations in the G glycoprotein:

- **S175T:** Located in the neck region between the head and stalk domains, this site overlaps a linear T-cell epitope (aa 164–178) previously identified in the Nipah virus (NiV) G [24], where threonine is also present. Although the substitution is conservative, its presence in an immunogenic region raises the possibility that it could modulate antigenicity and contribute to immune evasion. This substitution is prevalent in Clade D, being present in strain 71, used in our in vitro assays (ARTOW003_VTM_u_52_1).

- **A336T:** This substitution is located at a solvent-exposed surface of the head domain. Structural analysis reveals that A336T is distant from the receptor-binding interface and, based on superimposition of HeV G structures with cryo-EM models of the Nipah virus G tetramer (PDB ids: 8K0C and 8K0D) [20,25,26] in the compact and loose conformations, it is also positioned away from known oligomerization interfaces. Currently, there is no evidence supporting a functional role for this substitution. However, it is notable that a threonine is present at the equivalent position in the closely related HeV-g2 lineage. This conservation across distinct genetic backgrounds may suggest that the substitution is tolerated without detrimental effects on viral fitness or could reflect subtle lineage-specific structural adaptations worth investigating further. This substitution is Clade C-defining and is present in all Clade D strains, including strain 71, used in our in vitro assays (ARTOW003_VTM_u_52_1).

In addition to LD variants, we identified other substitutions of potential functional relevance, despite having a single occurrence in our dataset:

- **T373I:** A sub-clade-defining mutation in clade B, this residue lies adjacent to a conserved, immunodominant T-cell epitope described in NiV (aa. 360-370) [24]. Given its proximity, T373I may alter epitope presentation or processing, thereby contributing to immune escape in clade B genotypes. This substitution is found in Clade B, strains ARTOW005_AVL_U_16_1, ARTOW006_AVL_U_6_1, and ARTOW006_VTM_U_14_1 (strain 84).
- **R248G:** This substitution represents a chemically drastic change within the head domain, replacing a charged arginine with glycine. Structural modeling suggests that R248 may contribute to local secondary structure stability and electrostatic interactions, including a salt bridge (Fig. 4a). Notably, K246, located nearby, has been shown to become exposed upon receptor binding and plays a critical role in F triggering [20]. Based on spatial proximity and structural context, we hypothesize that R248G could affect syncytium formation and the efficiency of fusion triggering.

There is only one occurrence of this mutation in our genomic dataset. This substitution is found in Clade C, strain ARRED004_AVL_U_23_1.

- **N306K:** This residue lies is a known N-linked glycosylation site, and prior mutagenesis studies in HeV G have shown that disrupting glycosylation at this site (via N-to-Q substitution) leads to enhanced membrane fusion [27]. Therefore, N306K could increase G-mediated fusion activity and potentially alter viral spread. This substitution is found in B:ARCLU007_AVL_U_43_1.

2.5. RNA-dependent RNA polymerase (L)

The HeV L protein is the catalytic subunit of the viral RNA-dependent RNA polymerase complex and plays a central role in both transcription and replication of the viral genome, acting in concert with P, which stabilizes and positions L on the nucleocapsid. The HeV L protein is a multifunctional enzyme comprising several conserved domains, including the RNA-dependent RNA polymerase (RdRp) and domains responsible for cap addition (PRNTase or CAP), cap methylation (MTase), and polyadenylation. Mutations in L can influence polymerase processivity, RNA editing at the P gene, and interactions with other viral or host factors, potentially altering virulence and host adaptation.

The L protein is highly conserved between HeV and NiV, consistent with its critical function, but because of its large size, most LD substitutions are in L in our dataset. Based on structural comparison with the recently resolved structure of NiV L-P complex in the apo (PDB id.: 9GJT) and RNA-bound (PDB id.: 9GJU) [28] forms and using an AlphaFold-predicted model of HeV L, we evaluated the potential functional effects of the nine LD mutations in L protein found in our dataset. Most LD substitutions are likely silent, except by three of them. Interestingly, five of the nine LD substitutions in L result in amino acids that match those found in the reference sequences of NiV and/or HeV-g2, suggesting functional tolerance or convergence. Below, we summarize the structural context and potential significance of each variant:

- **Q141R:** Located on the solvent-exposed surface of the N-terminal domain (NTD), this substitution occurs in a region lacking known catalytic or interaction roles. Notably, NiV L

encodes an arginine at this position, suggesting functional compatibility. This substitution is prevalent in Clade D.

- **V215I:** A conservative substitution buried within the NTD, this site is not part of any recognized functional motif. It mirrors the residue found in NiV L, further supporting its likely neutrality. It is another case of substitution leading to the corresponding amino acid in NiV L. This substitution is prevalent in Clade B.
- **S260P:** Located in the NTD, this substitution replaces a serine with a proline, potentially affecting local backbone flexibility. While not in a defined catalytic region, proline-induced rigidity could alter structural dynamics. NiV L also encodes proline at this position. This substitution is Clade D-defining.
- **Q321H:** Positioned near the putative nucleoside triphosphate entry channel and adjacent to the region that interacts with the XD domain of the P protein, this substitution introduces a positively charged histidine. This could theoretically modulate the electrostatic environment of the entry channel and influence NTP affinity (Fig. 4c). While speculative, this site may warrant further functional investigation. This substitution is Clade C-defining and is prevalent in Clade D including strain 71, used in our in vitro assays (ARTOW003_VTM_u_52_1).
- **M645T** and **K658R**: These sites lie within a unique, flexible insertion in the palm subdomain (residues 603–711), which lacks resolved density in the NiV L structure and is absent in most other mononegaviruses, having undetermined function. K658R preserves the positive charge and is likely functionally silent. The substitution M645T is Clade A-defining and K658R is Clade D-defining.
- **A1655T:** This site is located in the connector domain (CD), which becomes ordered upon RNA binding. The substitution introduces threonine—the residue also found in NiV L and HeV-g2—suggesting functional tolerance. No known motifs are affected. This substitution is Clade B-defining.

- **H2081R:** Positioned on the surface of the methyltransferase (MTase) domain, this substitution is near conserved regions involved in RNA modification, including the KKG motif. Although it maintains a positively charged residue, potential indirect effects on RNA coordination or domain electrostatics cannot be ruled out. The substitution converges to the amino acid in HeV-g2. This substitution is Clade A-defining.
- **I2110T:** Located within the C-terminal domain (CTD), this residue contributes to hydrophobic contacts with the helix containing the KKG motif, a region implicated in mRNA capping and methylation. The introduction of threonine could perturb local packing, potentially affecting CTD flexibility or RNA interaction indirectly. This substitution is Clade A-defining.

Supplementary Tables

Supplementary Table 1: List of flying fox roost sites at which sampling was conducted in Queensland (Qld) and New South Wales (NSW) Australia, their latitude and longitude (rounded to 2 decimal places), the number of samples screened, their HeV-g1 result (where positive is Ct ≤40), and the number of genomes recovered in total and across each clade. The name and location of one site is anonymized for privacy reasons.

Roost site	Lat	Long	Total samples screened	HeV-g1 positive	HeV-g1 negative	Total Genomes	Clade A	Clade B	Clade C	Clade D
Bundaberg, Qld	-24.87	152.37	66	3	63					
Hervey Bay, Qld	-25.29	152.89	191	11	180	1				1
Gympie (Commissioner's Gully), Qld	-26.18	152.66	58	6	52					
Gympie (Township), Qld	-26.18	152.64	71	5	66					
Redcliffe, Qld	-27.23	153.1	1927	126	1801	5	1	1	2	1
Mount Ommaney, Qld	-27.54	152.93	86	9	77	1				1
Sunnybank, Qld	-27.58	153.05	1368	67	1301	3		1		2
Toowoomba, Qld	-27.6	151.94	1914	121	1793	17	2	4	2	9
Canungra, Qld	-28.04	153.18	261	11	250	1	1			
Burleigh, Qld	-28.08	153.44	632	23	609					
Currumbin, Qld	-28.15	153.47	53	8	45	1		1		
Anchorage, NSW	-28.19	153.53	50	3	47					
Banora Green, NSW	-28.22	153.54	32	5	27					
Tyalgum, NSW	-28.35	153.2	57	2	55					
Stokers Siding, NSW	-28.4	153.41	63	5	58					
Mullumbimby, NSW	-28.55	153.49	44	6	38					
Simpson's Creek, NSW	-28.64	153.58	97	20	77	2			2	
Byron Bay, NSW	-28.64	153.62	45	2	43					
Doroughby, NSW	-28.68	153.36	59	1	58					
Clunes, NSW	-28.73	153.42	1788	202	1586	12	3	4		5
Lismore, NSW	-28.81	153.3	364	31	333	2			1	1
Maclean, NSW	-29.47	153.2	122	0	122					
Nambucca Heads, NSW	-30.64	153	315	36	279	3	1		2	
Stewarts Brook, NSW	-32	151.23	174	0	174					
IP	NA	NA	32	0	32					
**TOTAL**			**9869**	**703**	**9166**	**48**	**8**	**11**	**9**	**20**

Supplementary Table 2. Regions and corresponding latitudes/longitudes used for discrete transition reconstruction.

Site	Region	Region average latitude	Region average longitude
Brisbane	Brisbane - Southeast Qld	-27.55	153.13
Canungra	Brisbane - Southeast Qld	-27.55	153.13
Cedar_Grove	Brisbane - Southeast Qld	-27.55	153.13
Currumbin	Brisbane - Southeast Qld	-27.55	153.13
Currumbin_Valley	Brisbane - Southeast Qld	-27.55	153.13
Mount_Ommaney	Brisbane - Southeast Qld	-27.55	153.13
Peachester	Brisbane - Southeast Qld	-27.55	153.13
Redcliffe	Brisbane - Southeast Qld	-27.55	153.13
Redlands	Brisbane - Southeast Qld	-27.55	153.13
Sunnybank	Brisbane - Southeast Qld	-27.55	153.13
Tamborine_Mt	Brisbane - Southeast Qld	-27.55	153.13
Clunes	Lismore-Byron-Ballina	-28.71	153.44
Lismore	Lismore-Byron-Ballina	-28.71	153.44
Mullumbimby	Lismore-Byron-Ballina	-28.71	153.44
Murwillumbah	Lismore-Byron-Ballina	-28.71	153.44
Newrybar	Lismore-Byron-Ballina	-28.71	153.44
Pimlico	Lismore-Byron-Ballina	-28.71	153.44
Simpsons_Creek	Lismore-Byron-Ballina	-28.71	153.44
Tintenbar	Lismore-Byron-Ballina	-28.71	153.44
Nambucca_Heads	Mid coast NSW	-30.65	152.93
South_Arm	Mid coast NSW	-30.65	152.93
Cawarral	North and Central Qld	-20.26	148.33
Clifton_Beach	North and Central Qld	-20.26	148.33
Mackay	North and Central Qld	-20.26	148.33
Proserpine	North and Central Qld	-20.26	148.33
Tolga	North and Central Qld	-20.26	148.33
Yeppoon	North and Central Qld	-20.26	148.33
Hervey_Bay	Southeast Qld	-25.11	152.57
South_Kolan	Southeast Qld	-25.11	152.57
Toowoomba	Toowoomba	-27.60	151.94
Cardiff_Heights	Cardiff_Heights		

Supplementary Table 3. Bayes-factor and posterior-probability for discrete reconstruction transition.

**From**	**To**	**Bayes-factor**	**Posterior-probability**
Southeast_Qld	Brisbane_Southeast_Qld	9616.78	1
Southeast_Qld	Toowoomba	491.14	1
Southeast_Qld	North_Central_Qld	20.71	0.91
Southeast_Qld	Mid_coast_NSW	8.84	0.81
Lismore_Byron_Ballina	Brisbane_Southeast_Qld	6.36	0.75
Brisbane_Southeast_Qld	North_Central_Qld	5.87	0.73
Southeast_Qld	Cardiff_Heights	2.51	0.54
Brisbane_Southeast_Qld	Toowoomba	1.88	0.47
Brisbane_Southeast_Qld	Cardiff_Heights	1.38	0.39
Brisbane_Southeast_Qld	Mid_coast_NSW	0.57	0.21
North_Central_Qld	Toowoomba	0.54	0.2
Lismore_Byron_Ballina	North_Central_Qld	0.32	0.13
Southeast_Qld	Lismore_Byron_Ballina	0.28	0.11
Cardiff_Heights	Toowoomba	0.28	0.12
North_Central_Qld	Cardiff_Heights	0.25	0.1
Lismore_Byron_Ballina	Cardiff_Heights	0.17	0.07
Mid_coast_NSW	North_Central_Qld	0.17	0.07
Lismore_Byron_Ballina	Mid_coast_NSW	0.15	0.06
Lismore_Byron_Ballina	Toowoomba	0.13	0.06
Mid_coast_NSW	Cardiff_Heights	0.12	0.05
Mid_coast_NSW	Toowoomba	0.11	0.05

Supplementary Table 4. Model selection for HeV-g1 genome (codon) dataset

Model	PS-logML	SS-logML	PS-Rank	SS-Rank	PS-BF	SS-BF
Relaxed, SkyGrid	-27932.3	-27932.88	1	1	0	0
Relaxed, Constant	-27936.61	-27936.73	2	2	4.3	3.84
Strict, SkyGrid	-27938.79	-27939.06	3	3	6.48	6.18
Strict, Constant	-27942.08	-27942.06	4	4	9.77	9.18

#PS=Path sampling, SS= Stepping-stone sampling, BF=Bayes Factor

Supplementary Table 5: Lineage-defining amino acid substitutions in HeV and the corresponding residues in HeV-g2, NiV-Bangladesh (NiV_{BD}), and NiV-Malaysia (NiV_{MY}). Convergent substitutions observed across strains are highlighted in red.

**Protein**	**P/V/W**					**G**	
**HeV**	R149C	G219E	D304G	K317E	P352S	S175T	A336T
**HeV-g2**	C149	G219	G304	K317	P352	S174	T335
**NiV_{BD}**	C149	E219	G308	P321	I352	T175	A336
**NiV_{MY}**	C149	E219	G308	P321	I352	T175	A336

**Protein**	**L**								
**HeV**	Q141R	V215I	S260P	Q321H	M645T	K658R	A1655T	H2081R	I2110T
**HeV-g2**	Q141	I215	P260	Q321	M645	R658	T1655	R2081	V2110
**NiV_{BD}**	R141	I215	P260	Q321	K645	Y658	T1655	K2081	I2110
**NiV_{MY}**	R141	I215	P260	Q321	K645	H658	T1655	K2081	I2110

Supplementary Table 6: Long range PCR assay (primers, cycling program and reaction mix).

**Primers**					
**Name**	**Sequence**	**Position**	**Tm**	**%GC**	**nt**
HeV_G_F1	TAGGACCCAAGTCCTTAACCACATTCTA	8679	68.1	42.9	28
HeV_G_F2	GATCTAAAACTAGTATGATGGCTGATTCCAA	8899	67.5	35.5	31
HeV_G_RB	GCTCTTTTGGTCAATCAACTCTCTGA	10741	67.8	42.3	26

**Reaction mix**				
PrimeSTAR GXL Buff	5X	10	uL	40
dNTP Mix	2.5mM	4	uL	16
primer 1	10uM	1	uL	4
primer 2	10uM	1	uL	4
Template cDNA	UNK	5	uL	NA
PrimeSTAR GXL Polymerse	1.25U/uL	1	uL	4
PCR grade H2O	NA	28		112
	total	50	uL	

**Reaction condition**				
Thermal cycling	(°C)	Time		
step 1		98	2:00	
step 2		98	0:10	
step 3		66	0:15	(-2C per cycle)
step 4		72	10:00	
step 5	got to step 2 four times			

step 6		98	0:10
step 7		56	0:15
step 8		72	10:00
step 9	go to step 6 thirty-one times		
step 10		4	forever

Supplementary Table 7: Host genes used RT-qPCR

Gene (primer) name	Sequence
Human_*Ifnb1* F	CAGCAGTTCCAGAAGGAGGA
Human_ *Ifnb1* R	AGCCAGGAGGTTCTCAACAA
Human_ *Ifnb1* P	FAM-CGCCGCATTGACCATCTATGAGATGC
Human_*Il6* F	ATGCCAGCCTGCTGACGAAG
Human_*Il6* R	AAGAGCCCTCAGGCTGGACT
Human_*Il6* P	FAM-CCTGCAGCCACTGGTTCTGTGCCTGC
Human_*Ifit1* F	GGGCAACTTTGCCTGGATGT
Human_*Ifit1* R	GCAAGGCCCATCCTTCCTCA
Human_*Ifit1* P	FAM-ACCACATGGGCAGACTGGCAGAAGCCC
Human_Mx1 F	AGCGCATCTCCAGCCACATC
Human_Mx1 R	GTGTCGCTCCGCTCCTTCAG
Human_Mx1 P	FAM-AGGCCATGCTGCAGCTCCTGCAGGA
Black flying fox_ *Ifnb1* F	AGCACTGGCTGGAATGAAACCA
Black flying fox_ *Ifnb1* R	GCAGCACTGTCATGCTTTCCC
Black flying fox_ *Ifnb1* P	FAM-TCCAGACGGTCCATCTGCCCACCGAGT
Black flying fox_*Il6* F	AGGCGATGCAGATGCGAACC
Black flying fox_*Il6* R	TCGTGGTCTGCAGCCATTCG
Black flying fox_*Il6* P	FAM-AGCCTGCTGGCTAAGCTGCAGTCGCA
Black flying fox_*Ifit1* F	CATGGGCAGCCTGGGAGAAG
Black flying fox_*Ifit1* R	AAAGCAGGCCTTGGCTCGTT
Black flying fox_*Ifit1* P	FAM-TGCAAGAAGCTTGCCAGTCCCTCCTGC
Black flying fox_*Mx1* F	TGGTGGTGCCCAGTAACGTG
Black flying fox_*Mx1* R	AGGTCGGGCTTCGTCAGGAT
Black flying fox_*Mx1* P	FAM-TCGCCACCACGGAGGCCTTGAGCA
Horse_ *Ifnb1* F	ACACAACCATTCTGCGCCTGA
Horse_ *Ifnb1* R	GGAAGGCCAAGTTCCTGAGCA
Horse_ *Ifnb1* P	FAM-CCGCTTGGACCACTGTCCAGGCACAGT
Horse_*Il6* F	CCTCTTCACAAGCACCGTCA
Horse_*Il6* R	TCTTCTCCCAGGGGTAGTGG
Horse_*Il6* P	FAM-AGTTGCCTTCTCCCTGGGGCTGCT
Horse_*Ifit1* F	AGGTTCTCCTTGCCCTGAAGC
Horse_*Ifit1* R	TGGCCGCATATCGAAGGACA
Horse_*Ifit1* P	FAM-AGCGCTGACCAACACGTCCTCGCAGA
Horse_*Mx1* F	AGCATGGCTCAGGAGGTGGA
Horse_*Mx1* R	TCGGTCCTGGATGTCCTGCT
Horse_*Mx1* P	FAM-ACGTCTACCACCTGCTCCTCGGTGCCT
Hprt F	AGATGGTCAAGGTCGCAAG
Hprt R	CCTGAAGTATTCATTATAGTCAAGGG
Hprt P	FAM-ACTTTGTTGGATTTGAAATTCCAGACAAGTTTG

Ifnb = Interferon beta, *Il* = Interleukin, *Ifit* = Interferon-induced protein with tetratricopeptide, Mx = myxovirus resistance, *Hprt* = hypoxanthine phosphoribosyltransferase, F = forward primer, reverse primer, P = Probe.

Supplementary Table 8. Positive selection in HeV-g1genome (codon) dataset

Site	Gene	Codon	FUBAR	MEME	SLAC
2882	L	141	+	-	-

P values of <0.05 or posterior probability values of >0.95 were considered to provide significant evidence of positive selection.

Supplementary Figures

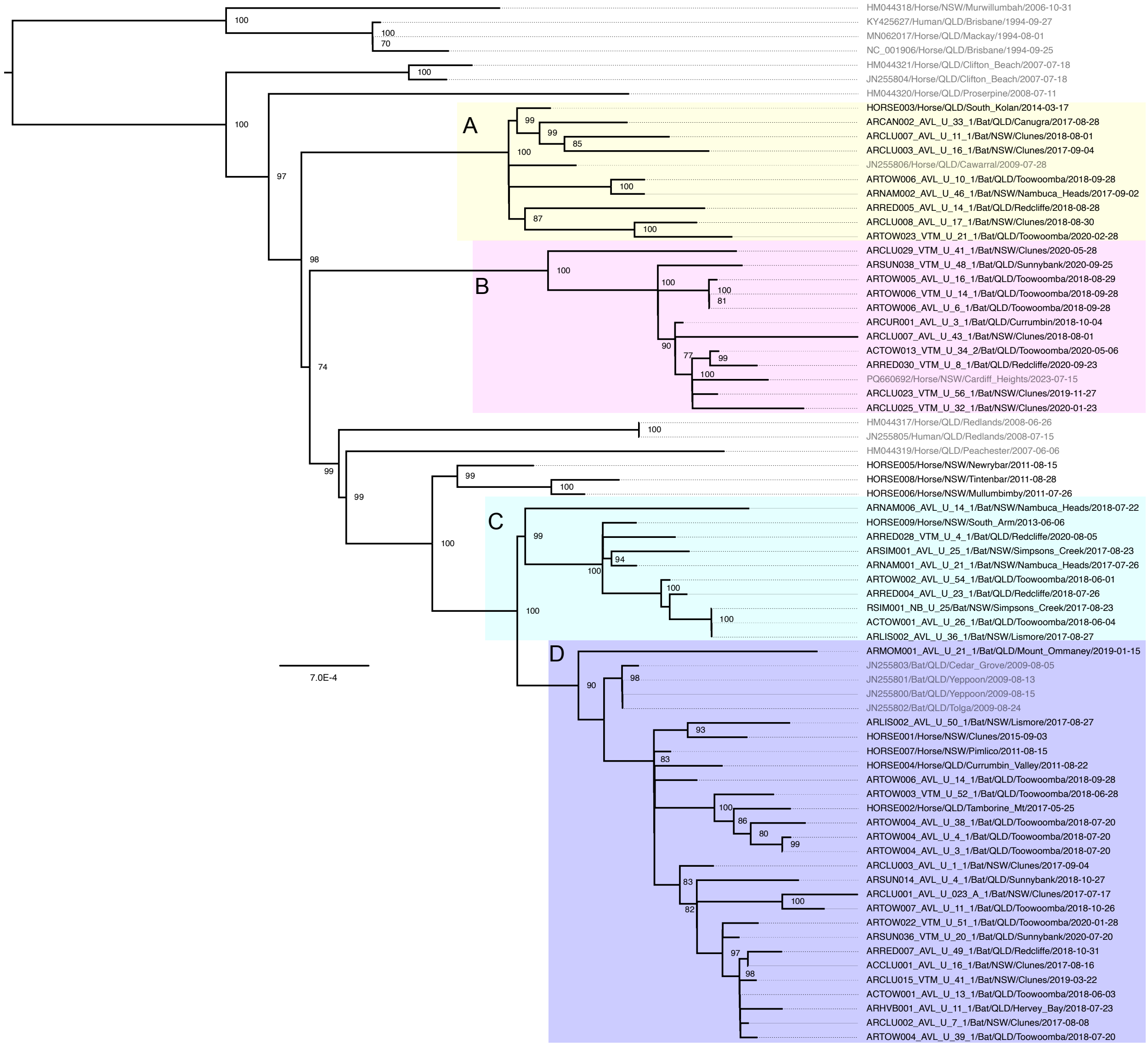

Supplementary Fig. 1: Coding sequence phylogeny of HeV-g1. Of the 73 genomes used in the phylogeny, 57 were newly generated in this study. The alignment was built with MAFFT (FFT-NS-1 algorithm [29], with the best model for distance estimates (TN+F+I) identified with the ModelFinder function [30] as the one with the lowest Bayesian information criterion (BIC). Maximum likelihood phylogenetic tree was constructed using IG-TREE2 [31] and branch support was assessed using both ultrafast bootstrap approximation (ufBoot, 1000 replicates) [32] and SH-like approximate likelihood ratio test (SH-aLRT). The tree was visualized in FigTree (http://tree.bio.ed.ac.uk/software/figtree/), and midpoint rooted for purposes of clarity. Only bootstrap support values greater of 69 are shown. Bars indicate nucleotide substitutions per site. Greyed tips represent sequences obtained from GenBank.

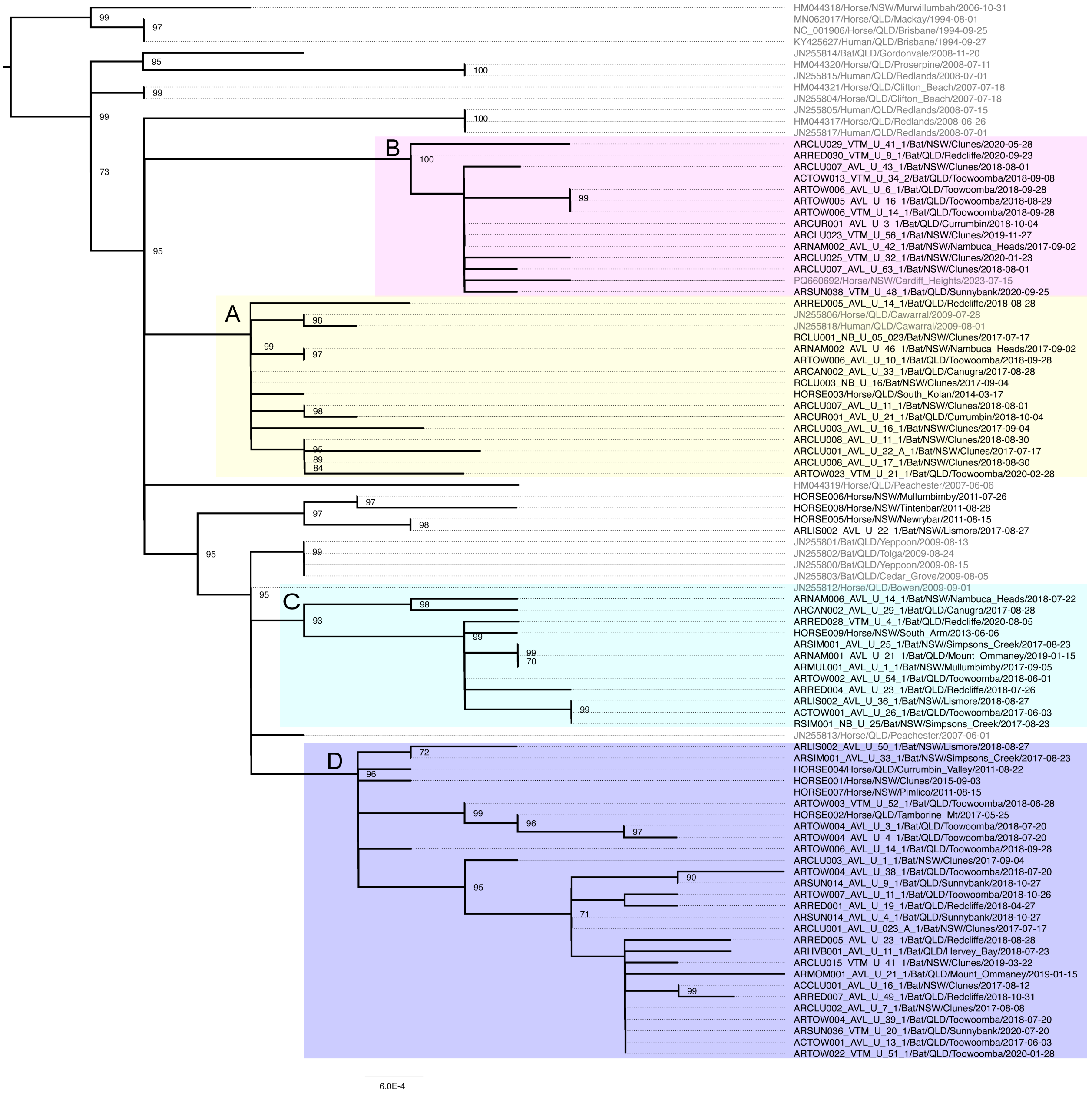

Supplementary Fig. 2: Glycoprotein G sequence phylogeny of HeV-g1. The alignment was built with MAFFT (FFT-NS-1 algorithm [29], and the best model for distance estimates (TN+F+I) identified with the ModelFinder function [30] as the one with the lowest Bayesian information criterion (BIC). Maximum likelihood phylogenetic tree was constructed using IG-TREE2 [31] and branch support was assessed using both ultrafast bootstrap approximation (ufBoot, 1000 replicates) [32] and SH-like approximate likelihood ratio test (SH-aLRT). The tree was visualized in FigTree (http://tree.bio.ed.ac.uk/software/figtree/), and midpoint rooted for purposes of clarity. Only bootstrap support values greater of 69 are shown. Bars indicate nucleotide substitutions per site. Greyed tips represent sequences obtained from GenBank.

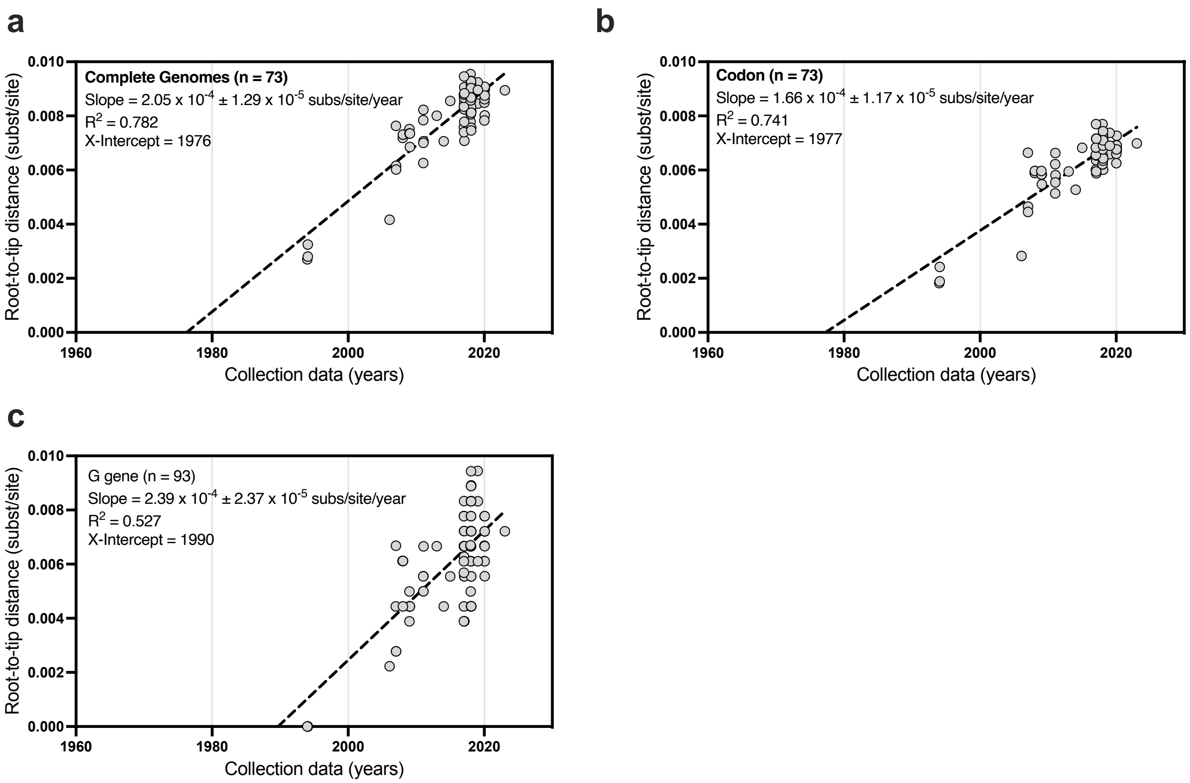

Supplementary Fig. 3: **Temporal signal in the HeV-g1 sequence data. (a) whole genome sequence, (b)** coding sequence**, (c)** complete G gene. The root-to-tip genetic distances from the ML phylogeny were plotted against sampling time, and a linear regression was performed to compare the clock-like structure.

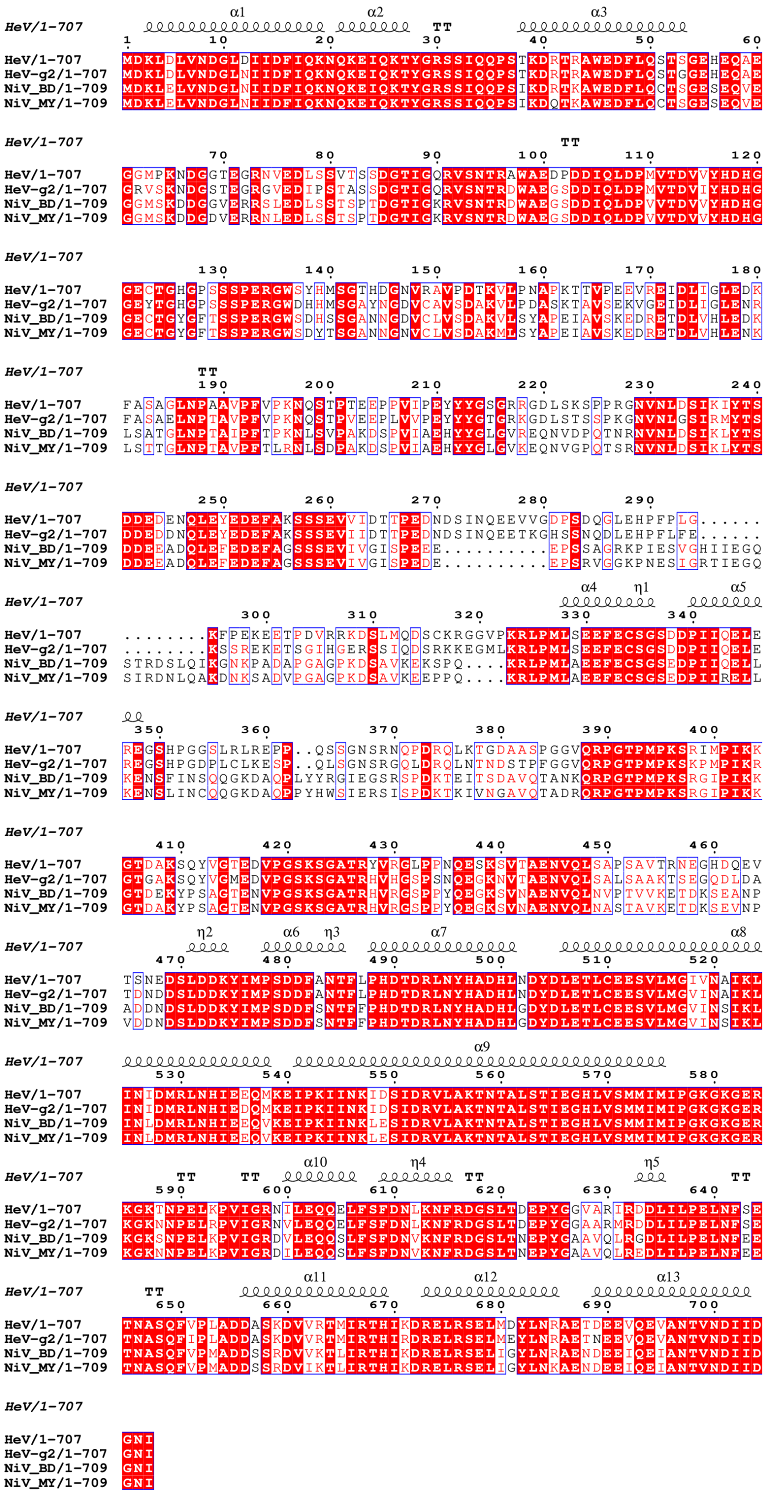

Supplementary Fig. 4: Sequence alignment of the phosphoprotein P protein from HeV, HeV-g2, NiV-Bangladesh (NiV_{BD}), and NiV-Malaysia (NiV_{MY}). The lineage-defining substitutions in P/V/W lie within their common long intrinsically disordered region (IDR), aa. 51-405. The substitution P352S, highlighted in this study, is lies near a highly conserved region within the IDR.

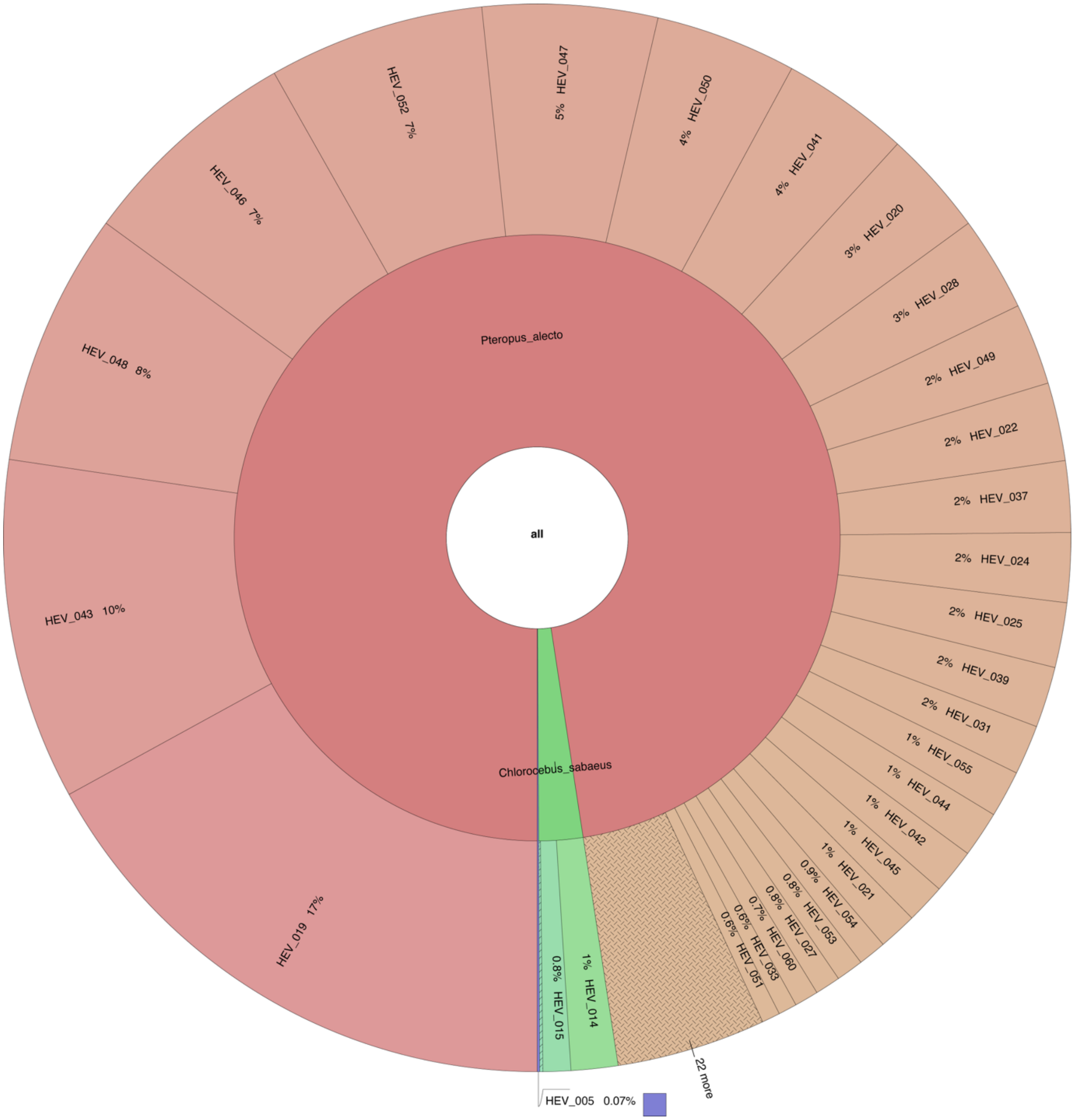

Supplementary Fig. 5: Krona visualization of host species assignment based on COX1 read alignment. Paired-end reads were trimmed for adapters and aligned to a Cytochrome Oxidase subunit 1 (COX1) reference database to verify field host species identity. The number of reads assigned to each species was summarized and divided by two to account for paired reads. Relative frequencies of COX1 reads per species were visualized using Krona, with the most abundant species (top hit) in each sample designated as the putative host.

References

1 Passchier, T. C. *et al.* The cryoEM structure of the Hendra henipavirus nucleoprotein reveals insights into paramyxoviral nucleocapsid architectures. *Scientific Reports* **14**, 14099 (2024). https://doi.org/10.1038/s41598-024-58243-z

2 Bourhis, J.-M. *et al.* Structural Dynamics of the C-terminal X Domain of Nipah and Hendra Viruses Controls the Attachment to the C-terminal Tail of the Nucleocapsid Protein. *Journal of Molecular Biology* **434**, 167551 (2022). https://doi.org/https://doi.org/10.1016/j.jmb.2022.167551

3 Shaw, M. L. Henipaviruses employ a multifaceted approach to evade the antiviral interferon response. *Viruses* **1**, 1190-1203 (2009). https://doi.org/10.3390/v1031190

4 Quarleri, J., Galvan, V. & Delpino, M. V. Henipaviruses: an expanding global public health concern? *Geroscience* **44**, 2447-2459 (2022). https://doi.org/10.1007/s11357-022-00670-9

5 Andrejeva, J. *et al.* The V proteins of paramyxoviruses bind the IFN-inducible RNA helicase, mda-5, and inhibit its activation of the IFN-beta promoter. *Proc Natl Acad Sci U S A* **101**, 17264-17269 (2004). https://doi.org/10.1073/pnas.0407639101

6 Sánchez-Aparicio, M. T., Feinman, L. J., García-Sastre, A. & Shaw, M. L. Paramyxovirus V Proteins Interact with the RIG-I/TRIM25 Regulatory Complex and Inhibit RIG-I Signaling. *J Virol* **92** (2018). https://doi.org/10.1128/jvi.01960-17

7 Mougari, S., Gonzalez, C., Reynard, O. & Horvat, B. Fruit bats as natural reservoir of highly pathogenic henipaviruses: balance between antiviral defense and viral toleranceInteractions between Henipaviruses and their natural host, fruit bats. *Curr Opin Virol* **54**, 101228 (2022). https://doi.org/10.1016/j.coviro.2022.101228

8 He, B. *et al.* Recovery of paramyxovirus simian virus 5 with a V protein lacking the conserved cysteine-rich domain: the multifunctional V protein blocks both interferon-beta induction and interferon signaling. *Virology* **303**, 15-32 (2002). https://doi.org/10.1006/viro.2002.1738

9 Atkinson, S. C. *et al.* Recognition by host nuclear transport proteins drives disorder-to-order transition in Hendra virus V. *Sci Rep* **8**, 358 (2018). https://doi.org/10.1038/s41598-017-18742-8

10 Rodriguez, J. J., Wang, L. F. & Horvath, C. M. Hendra virus V protein inhibits interferon signaling by preventing STAT1 and STAT2 nuclear accumulation. *J Virol* **77**, 11842-11845 (2003). https://doi.org/10.1128/jvi.77.21.11842-11845.2003

11 Becker, N. & Maisner, A. Nipah Virus Impairs Autocrine IFN Signaling by Sequestering STAT1 and STAT2 into Inclusion Bodies. *Viruses* **15** (2023). https://doi.org/10.3390/v15020554

12 Ludlow, L. E., Lo, M. K., Rodriguez, J. J., Rota, P. A. & Horvath, C. M. Henipavirus V protein association with Polo-like kinase reveals functional overlap with STAT1 binding and interferon evasion. *J Virol* **82**, 6259-6271 (2008). https://doi.org/10.1128/jvi.00409-08

13 Salladini, E. *et al.* Identification of a Region in the Common Amino-terminal Domain of Hendra Virus P, V, and W Proteins Responsible for Phase Transition and Amyloid Formation. *Biomolecules* **11** (2021). https://doi.org/10.3390/biom11091324

14 Blom, N., Gammeltoft, S. & Brunak, S. Sequence and structure-based prediction of eukaryotic protein phosphorylation sites. *J Mol Biol* **294**, 1351-1362 (1999). https://doi.org/10.1006/jmbi.1999.3310

15 Mariani, V., Biasini, M., Barbato, A. & Schwede, T. lDDT: a local superposition-free score for comparing protein structures and models using distance difference tests. *Bioinformatics* **29**, 2722-2728 (2013). https://doi.org/10.1093/bioinformatics/btt473

16 Abramson, J. *et al.* Accurate structure prediction of biomolecular interactions with AlphaFold 3. *Nature* **630**, 493-500 (2024). https://doi.org/10.1038/s41586-024-07487-w

17 Deffrasnes, C. *et al.* Genome-wide siRNA Screening at Biosafety Level 4 Reveals a Crucial Role for Fibrillarin in Henipavirus Infection. *PLOS Pathogens* **12**, e1005478 (2016). https://doi.org/10.1371/journal.ppat.1005478

18 Bauer, A. *et al.* ANP32B is a nuclear target of henipavirus M proteins. *PLoS One* **9**, e97233 (2014). https://doi.org/10.1371/journal.pone.0097233

19 Liu, Y. C., Grusovin, J. & Adams, T. E. Electrostatic Interactions between Hendra Virus Matrix Proteins Are Required for Efficient Virus-Like-Particle Assembly. *Journal of Virology* **92**, 10.1128/jvi.00143-00118 (2018). https://doi.org/doi:10.1128/jvi.00143-18

20 Fan, P. *et al.* A potent Henipavirus cross-neutralizing antibody reveals a dynamic fusion-triggering pattern of the G-tetramer. *Nat Commun* **15**, 4330 (2024). https://doi.org/10.1038/s41467-024-48601-w

21 Byrne, P. O. *et al.* Prefusion stabilization of the Hendra and Langya virus F proteins. *Journal of Virology* **98**, e01372-01323 (2024). https://doi.org/doi:10.1128/jvi.01372-23

22 Byrne, P. O. *et al.* Structural basis for antibody recognition of vulnerable epitopes on Nipah virus F protein. *Nature Communications* **14**, 1494 (2023). https://doi.org/10.1038/s41467-023-36995-y

23 Luque, L. E. & Russell, C. J. Spring-loaded heptad repeat residues regulate the expression and activation of paramyxovirus fusion protein. *J Virol* **81**, 3130-3141 (2007). https://doi.org/10.1128/jvi.02464-06

24 Huang, X. *et al.* Nipah virus attachment glycoprotein ectodomain delivered by type 5 adenovirus vector elicits broad immune response against NiV and HeV. *Front Cell Infect Microbiol* **13**, 1180344 (2023). https://doi.org/10.3389/fcimb.2023.1180344

25 Bowden, T. A. *et al.* Structural basis of Nipah and Hendra virus attachment to their cell-surface receptor ephrin-B2. *Nature Structural & Molecular Biology* **15**, 567-572 (2008). https://doi.org/10.1038/nsmb.1435

26 Bowden, T. A., Crispin, M., Harvey, D. J., Jones, E. Y. & Stuart, D. I. Dimeric Architecture of the Hendra Virus Attachment Glycoprotein: Evidence for a Conserved Mode of Assembly. *Journal of Virology* **84**, 6208-6217 (2010). https://doi.org/doi:10.1128/jvi.00317-10

27 Bradel-Tretheway, B. G., Liu, Q., Stone, J. A., McInally, S. & Aguilar, H. C. Novel Functions of Hendra Virus G N-Glycans and Comparisons to Nipah Virus. *Journal of Virology* **89**, 7235-7247 (2015). https://doi.org/doi:10.1128/jvi.00773-15

28 Sala, F. A., Ditter, K., Dybkov, O., Urlaub, H. & Hillen, H. S. Structural basis of Nipah virus RNA synthesis. *Nat Commun* **16**, 2261 (2025). https://doi.org/10.1038/s41467-025-57219-5

29 Katoh, K. & Standley, D. M. MAFFT multiple sequence alignment software version 7: improvements in performance and usability. *Mol Biol Evol* **30**, 772-780 (2013). https://doi.org/10.1093/molbev/mst010

30 Kalyaanamoorthy, S., Minh, B. Q., Wong, T. K. F., von Haeseler, A. & Jermiin, L. S. ModelFinder: fast model selection for accurate phylogenetic estimates. *Nat Methods* **14**, 587-589 (2017). https://doi.org/10.1038/nmeth.4285

31 Minh, B. Q. *et al.* IQ-TREE 2: New Models and Efficient Methods for Phylogenetic Inference in the Genomic Era. *Molecular Biology and Evolution* **37**, 1530-1534 (2020). https://doi.org/10.1093/molbev/msaa015

32 Hoang, D. T., Chernomor, O., von Haeseler, A., Minh, B. Q. & Vinh, L. S. UFBoot2: Improving the Ultrafast Bootstrap Approximation. *Mol Biol Evol* **35**, 518-522 (2018). https://doi.org/10.1093/molbev/msx281
