# Supplementary material for: Spatio-temporal dynamics of Hendra virus in Australia reveal stable maintenance of diverse viral clades among Pteropus bats
Source: Nat Microbiol. 2026 Apr 7;11(4):851–66. doi: 10.1038/s41564-025-02254-7 (PMC13056563; doi:10.1038/s41564-025-02254-7)
Supplement: Supplementary file 2 — Reporting Summary [file 41564_2025_2254_MOESM2_ESM.pdf]

nature portfolio

Corresponding author(s): Drs Vincent Munster and Alison Peel

Last updated by author(s): Nov 20, 2025

Reporting Summary

Nature Portfolio wishes to improve the reproducibility of the work that we publish. This form provides structure for consistency and transparency in reporting. For further information on Nature Portfolio policies, see our Editorial Policies and the Editorial Policy Checklist.

Statistics

For all statistical analyses, confirm that the following items are present in the figure legend, table legend, main text, or Methods section.

n/a	Confirmed	
☐	☒	The exact sample size (*n*) for each experimental group/condition, given as a discrete number and unit of measurement
☒	☐	A statement on whether measurements were taken from distinct samples or whether the same sample was measured repeatedly
☐	☒	The statistical test(s) used AND whether they are one- or two-sided<br>*Only common tests should be described solely by name; describe more complex techniques in the Methods section.*
☒	☐	A description of all covariates tested
☐	☒	A description of any assumptions or corrections, such as tests of normality and adjustment for multiple comparisons
☒	☐	A full description of the statistical parameters including central tendency (e.g. means) or other basic estimates (e.g. regression coefficient) AND variation (e.g. standard deviation) or associated estimates of uncertainty (e.g. confidence intervals)
☐	☒	For null hypothesis testing, the test statistic (e.g. *F*, *t*, *r*) with confidence intervals, effect sizes, degrees of freedom and *P* value noted<br>*Give P values as exact values whenever suitable.*
☐	☒	For Bayesian analysis, information on the choice of priors and Markov chain Monte Carlo settings
☒	☐	For hierarchical and complex designs, identification of the appropriate level for tests and full reporting of outcomes
☐	☒	Estimates of effect sizes (e.g. Cohen's *d*, Pearson's *r*), indicating how they were calculated

Our web collection on statistics for biologists contains articles on many of the points above.

Software and code

Policy information about availability of computer code

Data collection	NA
Data analysis	R 4.3.0 using the following packages; tidyverse, magrittr, maps, mapproj, ozmaps, sf, scatterpie, stringr ggnewscale, ggforce, and ggspatial<br>GraphPad Prism 10<br>IG-TREE2<br>MAFFT v7.505<br>JalView v10.0.5<br>TempEst v1.5.3<br>BEAST v1<br>FigTree v1.4.4<br>HyPhy package of datamonkey

For manuscripts utilizing custom algorithms or software that are central to the research but not yet described in published literature, software must be made available to editors and reviewers. We strongly encourage code deposition in a community repository (e.g. GitHub). See the Nature Portfolio guidelines for submitting code & software for further information.

Data

Policy information about availability of data

All manuscripts must include a data availability statement. This statement should provide the following information, where applicable:

- Accession codes, unique identifiers, or web links for publicly available datasets
- A description of any restrictions on data availability
- For clinical datasets or third party data, please ensure that the statement adheres to our policy

All novel sequences reported here have been submitted in GenBank (accession numbers are in Extended Data Table 2).

Research involving human participants, their data, or biological material

Policy information about studies with human participants or human data. See also policy information about sex, gender (identity/presentation), and sexual orientation and race, ethnicity and racism.

Reporting on sex and gender	Research does not involve human participants
Reporting on race, ethnicity, or other socially relevant groupings	Research does not involve human participants
Population characteristics	Research does not involve human participants
Recruitment	Research does not involve human participants
Ethics oversight	Research does not involve human participants

Note that full information on the approval of the study protocol must also be provided in the manuscript.

Field-specific reporting

Please select the one below that is the best fit for your research. If you are not sure, read the appropriate sections before making your selection.

☐ Life sciences ☐ Behavioural & social sciences ☒ Ecological, evolutionary & environmental sciences

For a reference copy of the document with all sections, see nature.com/documents/nr-reporting-summary-flat.pdf

Ecological, evolutionary & environmental sciences study design

All studies must disclose on these points even when the disclosure is negative.

Study description	This study aimed to enhance the understanding of Hendra virus (HeV) evolution and transmission by conducting extensive spatiotemporal sampling and whole-genome sequencing of HeV-positive samples from bats and horses.
Research sample	Sampling was conducted at flying fox roosts in southeast Queensland and mid- to north-coast New South Wales. Horse Hendra virus sequences included in this study came from horses infected with Hendra virus and then diagnosed and and samples from positive horses were sequenced at the Australian Centre for Disease Preparedness (ACDP).
Sampling strategy	Sample sizes were predetermined based on expected viral prevalence and statistical power calculations.
Data collection	December 2016 - September 2020. Data were collected via field sampling of bat populations, molecular viral screening assays and sequencing. Field samples were handled by trained field teams, with data collection occurring either on paper data sheets, transferred to digital formats, or directly onto a tablet data collection form.
Timing and spatial scale	Field data were collected monthly over four consecutive years at multiple roosting sites across southeastern Australia. Laboratory data were gathered continuously over the course of the study.
Data exclusions	We excluded one sequence with poor coverage.
Reproducibility	Because this is a field study in wild populations, results cannot be directly replicated. However, findings were replicated across sites and years.
Randomization	Individuals were captured in nets at the roost site. All captured individuals that produced a urine sample during holding or processing were included in screening. Multiple urine samples were pooled from each under-roost sheet, and a pooled sample from each sheet was screened. Samples selected for further analyses (including sequencing) were those with a Ct value <32.

Blinding	The laboratory team was blind to the metadata associated with each sample.

Did the study involve field work? ☒ Yes ☐ No

Field work, collection and transport

Field conditions	Fieldwork was conducted across all seasons and under varying climatic conditions, however rain-affected sampling sessions were abandoned and rescheduled.
Location	Sampling occurred in southeastern Australia, in roost sites located between 24.87°S and 32.0°S latitude . Full site details are provided in Supplementary Table 1
Access & import/export	Samples were exported from Australia to the USA. All necessary permits for sample collection were obtained from relevant wildlife and government authorities.
Disturbance	Disturbance to bat populations was minimized by conducting individual sampling outside of the birthing and early lactation season and limiting the time of interaction with captured animals.

Reporting for specific materials, systems and methods

We require information from authors about some types of materials, experimental systems and methods used in many studies. Here, indicate whether each material, system or method listed is relevant to your study. If you are not sure if a list item applies to your research, read the appropriate section before selecting a response.

Materials & experimental systems

n/a	Involved in the study	
☐	☒	Antibodies
☐	☒	Eukaryotic cell lines
☒	☐	Palaeontology and archaeology
☐	☒	Animals and other organisms
☒	☐	Clinical data
☒	☐	Dual use research of concern
☒	☐	Plants

Methods

n/a	Involved in the study	
☒	☐	ChIP-seq
☒	☐	Flow cytometry
☒	☐	MRI-based neuroimaging

Antibodies

Antibodies used	pSTAT1 – Y701 (Cell Signaling Technology, 9167S)<br>pSTAT2 – Y690 (Cell Signaling Technology, 88410S)<br>total STAT1 (Cell Signaling Technology, 14994S)<br>total STAT2 (Cell Signaling Technology, 72604S)<br>pTBK1-172 (Cell Signaling Technology, 5483T)<br>Actin (GeneTex, GTX629630)<br>Donkey-anti-rabbit (GE Healthcare, NA934)<br>Sheep-anti-mouse (GE Healthcare, NA931)
Validation	All of the above antibodies were validated by the manufacturer via western blot, flow cytometry, and/or immunohistochemistry

Eukaryotic cell lines

Policy information about cell lines and Sex and Gender in Research

Cell line source(s)	HFL-1, NBL-6 and Vero E6 cells were sourced from ATCC.<br>PaKiT cells were sourced from Michelle Baker at ACDP/AAHL in Australia.
Authentication	All cell lines were authenticated using Cyt sequencing.
Mycoplasma contamination	All cell lines were tested regularly for Mycoplasma contamination.
Commonly misidentified lines<br>(See ICLAC register)	No commonly misidentified cell lines were used.

Animals and other research organisms

Policy information about studies involving animals; ARRIVE guidelines recommended for reporting animal research, and Sex and Gender in Research

Laboratory animals	NA
Wild animals	Urine samples were collected from plastic sheets placed underneath flying fox roosts. Urine samples were also collected directly from individual bats captured in mist nests at their roost site. Bats were held in cotton bags with the bottom third lined with plastic and a urine collection bag attached to facilitate the collection of samples. Bats were anaesthetised for further sample collection and their species, sex, and age class (adult, subadult, juvenile) were recorded. Urine samples were collected directly from the bat if it urinated while under anaesthetic, or from the urine collection bag. Bats were released after sample collection.
Reporting on sex	Bats were anaesthetised for further sample collection and their species, sex, and age class (adult, subadult, juvenile) were recorded. However, the sequences analysis in this study does not include sex or age.
Field-collected samples	Urine samples from roosts or individual bats were kept frozen until extracted and screened for Hendra virus.
Ethics oversight	Griffith University Animal Ethics Committee Approval ENV/10/16/AEC and ENV/07/20/AEC. Personal Protective Equipment and disinfection protocols followed best practice guidelines (e.g. IUCN Bat Specialist Group, 2021; Wildlife Health Australia, 2020).

Note that full information on the approval of the study protocol must also be provided in the manuscript.

Plants

Seed stocks	No plant seed stocks were used.
Novel plant genotypes	No plants were used
Authentication	No plants were used
