# Supplementary material for: Spatio-temporal dynamics of Hendra virus in Australia reveal stable maintenance of diverse viral clades among Pteropus bats
Source: Nat Microbiol. 2026 Apr 7;11(4):851–66. doi: 10.1038/s41564-025-02254-7 (PMC13056563; doi:10.1038/s41564-025-02254-7)
Supplement: Supplementary file 4 — Metadata for sequences used in the analysis [file 41564_2025_2254_MOESM4_ESM.pdf]

Supplementary Data 1 | Metadata for sequences used in the analysis

ID	% of genomewith >2 reads	Ct	species	state	site	date	label	Clade
ACCLU001_AVL_U_16_1*	100.0	29	Bat	NSW	Clunes	8/12/17	ACCLU001_AVL_U_16_1/Bat/NSW/Clunes/2017-08-12	D
ACTOW001_AVL_U_13_1*	100.0	22	Bat	QLD	Toowoomba	6/3/17	ACTOW001_AVL_U_13_1/Bat/QLD/Toowoomba/2017-06-03	D
ACTOW001_AVL_U_26_1*	100.0	21	Bat	QLD	Toowoomba	6/3/17	ACTOW001_AVL_U_26_1/Bat/QLD/Toowoomba/2017-06-03	C
ACTOW013_VTM_u_34_2*	89.1	31	Bat	QLD	Toowoomba	9/8/18	ACTOW013_VTM_U_34_2/Bat/QLD/Toowoomba/2018-09-08	B
ARCAN002_AVL_U_33_1	99.5	29	Bat	QLD	Canugra	8/28/17	ARCAN002_AVL_U_33_1/Bat/QLD/Canugra/2017-08-28	A
ARCLU001_AVL_U_023_A	89.5	29	Bat	NSW	Clunes	7/17/17	ARCLU001_AVL_U_023_A_1/Bat/NSW/Clunes/2017-07-17	D
ARCLU002_AVL_U_7_1	100.0	29	Bat	NSW	Clunes	8/8/17	ARCLU002_AVL_U_7_1/Bat/NSW/Clunes/2017-08-08	D
ARCLU003_AVL_U_1_1	100.0	26	Bat	NSW	Clunes	9/4/17	ARCLU003_AVL_U_1_1/Bat/NSW/Clunes/2017-09-04	D
ARCLU003_AVL_U_16_1	98.7	28	Bat	NSW	Clunes	9/4/17	ARCLU003_AVL_U_16_1/Bat/NSW/Clunes/2017-09-04	A
ARCLU007_AVL_U_11_1	100.0	25	Bat	NSW	Clunes	8/1/18	ARCLU007_AVL_U_11_1/Bat/NSW/Clunes/2018-08-01	A
ARCLU007_AVL_U_43_1	93.0	29	Bat	NSW	Clunes	8/1/18	ARCLU007_AVL_U_43_1/Bat/NSW/Clunes/2018-08-01	B
ARCLU008_AVL_U_17_1	86.3	30	Bat	NSW	Clunes	8/30/18	ARCLU008_AVL_U_17_1/Bat/NSW/Clunes/2018-08-30	A
ARCLU015_VTM_U_41_1	98.4	29	Bat	NSW	Clunes	3/22/19	ARCLU015_VTM_U_41_1/Bat/NSW/Clunes/2019-03-22	D
ARCLU023_VTM_u_56_1	99.7	29	Bat	NSW	Clunes	11/27/19	ARCLU023_VTM_U_56_1/Bat/NSW/Clunes/2019-11-27	B
ARCLU025_VTM_u_32_1	97.8	30	Bat	NSW	Clunes	1/23/20	ARCLU025_VTM_U_32_1/Bat/NSW/Clunes/2020-01-23	B
ARCLU029_VTM_u_41_1	87.9	32	Bat	NSW	Clunes	5/28/20	ARCLU029_VTM_U_41_1/Bat/NSW/Clunes/2020-05-28	B
ARCUR001_AVL_U_3_1	100.0	27	Bat	QLD	Currumbin	10/4/18	ARCUR001_AVL_U_3_1/Bat/QLD/Currumbin/2018-10-04	B
ARHVB001_AVL_U_11_1	99.7	25	Bat	QLD	Hervey_Bay	7/23/18	ARHVB001_AVL_U_11_1/Bat/QLD/Hervey_Bay/2018-07-23	D
ARLIS002_AVL_U_36_1	100.0	28	Bat	NSW	Lismore	8/27/18	ARLIS002_AVL_U_36_1/Bat/NSW/Lismore/2018-08-27	C
ARLIS002_AVL_U_50_1	99.7	23	Bat	NSW	Lismore	8/27/18	ARLIS002_AVL_U_50_1/Bat/NSW/Lismore/2018-08-27	D
ARMOM001_AVL_U_21_1	99.6	30	Bat	QLD	Mount_Ommaney	1/15/19	ARMOM001_AVL_U_21_1/Bat/QLD/Mount_Ommaney/2019-01-15	D
ARNAM001_AVL_U_21_1	99.9	30	Bat	QLD	Nambuca_Heads	7/26/17	ARNAM001_AVL_U_21_1/Bat/NSW/Nambuca_Heads/2017-07-26	C
ARNAM002_AVL_U_46_1	100.0	30	Bat	NSW	Nambuca_Heads	9/2/17	ARNAM002_AVL_U_46_1/Bat/NSW/Nambuca_Heads/2017-09-02	A
ARNAM006_AVL_U_14_1	99.6	28	Bat	NSW	Nambuca_Heads	7/22/18	ARNAM006_AVL_U_14_1/Bat/NSW/Nambuca_Heads/2018-07-22	C
ARRED004_AVL_U_23_1	99.5	30	Bat	QLD	Redcliffe	7/26/18	ARRED004_AVL_U_23_1/Bat/QLD/Redcliffe/2018-07-26	C
ARRED005_AVL_U_14_1	100.0	28	Bat	QLD	Redcliffe	8/28/18	ARRED005_AVL_U_14_1/Bat/QLD/Redcliffe/2018-08-28	A
ARRED007_AVL_U_49_1	99.4	29	Bat	QLD	Redcliffe	10/31/18	ARRED007_AVL_U_49_1/Bat/QLD/Redcliffe/2018-10-31	D
ARRED028_VTM_u_4_1	91.9	32	Bat	QLD	Redcliffe	8/5/20	ARRED028_VTM_U_4_1/Bat/QLD/Redcliffe/2020-08-05	C
ARRED030_VTM_u_8_1	88.2	31	Bat	QLD	Redcliffe	9/23/20	ARRED030_VTM_U_8_1/Bat/QLD/Redcliffe/2020-09-23	B
ARSIM001_AVL_U_25_1	97.9	24	Bat	NSW	Simpsons_Creek	8/23/17	ARSIM001_AVL_U_25_1/Bat/NSW/Simpsons_Creek/2017-08-23	C
ARSUN014_AVL_U_4_1	99.4	26	Bat	QLD	Sunnybank	10/27/18	ARSUN014_AVL_U_4_1/Bat/QLD/Sunnybank/2018-10-27	D
ARSUN036_VTM_u_20_1	99.5	27	Bat	QLD	Sunnybank	7/20/20	ARSUN036_VTM_U_20_1/Bat/QLD/Sunnybank/2020-07-20	D
ARSUN038_VTM_u_48_1	99.9	26	Bat	QLD	Sunnybank	9/25/20	ARSUN038_VTM_U_48_1/Bat/QLD/Sunnybank/2020-09-25	B
ARTOW002_AVL_U_54_1	100.0	22	Bat	QLD	Toowoomba	6/1/18	ARTOW002_AVL_U_54_1/Bat/QLD/Toowoomba/2018-06-01	C
ARTOW003_VTM_u_52_1	100.0	31	Bat	QLD	Toowoomba	6/28/18	ARTOW003_VTM_U_52_1/Bat/QLD/Toowoomba/2018-06-28	D
ARTOW004_AVL_U_3_1	99.4	30	Bat	QLD	Toowoomba	7/20/18	ARTOW004_AVL_U_3_1/Bat/QLD/Toowoomba/2018-07-20	D

ARTOW004_AVL_U_38_1	96.6	29	Bat	QLD	Toowoomba	7/20/18	ARTOW004_AVL_U_38_1/Bat/QLD/Toowoomba/2018-07-20	D
ARTOW004_AVL_U_39_1	99.0	30	Bat	QLD	Toowoomba	7/20/18	ARTOW004_AVL_U_39_1/Bat/QLD/Toowoomba/2018-07-20	D
ARTOW004_AVL_U_4_1	94.1	31	Bat	QLD	Toowoomba	7/20/18	ARTOW004_AVL_U_4_1/Bat/QLD/Toowoomba/2018-07-20	D
ARTOW005_AVL_U_16_1	100.0	27	Bat	QLD	Toowoomba	8/29/18	ARTOW005_AVL_U_16_1/Bat/QLD/Toowoomba/2018-08-29	B
ARTOW006_AVL_U_10_1	100.0	25	Bat	QLD	Toowoomba	9/28/18	ARTOW006_AVL_U_10_1/Bat/QLD/Toowoomba/2018-09-28	A
ARTOW006_AVL_U_14_1	100.0	24	Bat	QLD	Toowoomba	9/28/18	ARTOW006_AVL_U_14_1/Bat/QLD/Toowoomba/2018-09-28	D
ARTOW006_AVL_U_6_1	100.0	26	Bat	QLD	Toowoomba	9/28/18	ARTOW006_AVL_U_6_1/Bat/QLD/Toowoomba/2018-09-28	B
ARTOW006_VTM_u_14_1	100.0	24	Bat	QLD	Toowoomba	9/28/18	ARTOW006_VTM_U_14_1/Bat/QLD/Toowoomba/2018-09-28	B
ARTOW007_AVL_U_11_1	100.0	25	Bat	QLD	Toowoomba	10/26/18	ARTOW007_AVL_U_11_1/Bat/QLD/Toowoomba/2018-10-26	D
ARTOW022_VTM_u_51_1	93.3	31	Bat	QLD	Toowoomba	1/28/20	ARTOW022_VTM_U_51_1/Bat/QLD/Toowoomba/2020-01-28	D
ARTOW023_VTM_u_21_1	92.5	30	Bat	QLD	Toowoomba	2/28/20	ARTOW023_VTM_U_21_1/Bat/QLD/Toowoomba/2020-02-28	A
HM044317	NA	NA	Horse	QLD	Redlands	6/26/08	HM044317/Horse/QLD/Redlands/2008-06-26	U
HM044318	NA	NA	Horse	NSW	Murwillumbah	10/31/06	HM044318/Horse/NSW/Murwillumbah/2006-10-31	U
HM044319	NA	NA	Horse	QLD	Peachester	6/6/07	HM044319/Horse/QLD/Peachester/2007-06-06	U
HM044320	NA	NA	Horse	QLD	Proserpine	7/11/08	HM044320/Horse/QLD/Proserpine/2008-07-11	U
HM044321	NA	NA	Horse	QLD	Clifton_Beach	7/18/07	HM044321/Horse/QLD/Clifton_Beach/2007-07-18	U
HORSE001	NA	18.6	Horse	NSW	Clunes	9/3/15	HORSE001/Horse/NSW/Clunes/2015-09-03	D
HORSE002	NA	14.5	Horse	QLD	Tamborine_Mt	5/25/17	HORSE002/Horse/QLD/Tamborine_Mt/2017-05-25	D
HORSE003	NA	15.6	Horse	QLD	South_Kolan	3/17/14	HORSE003/Horse/QLD/South_Kolan/2014-03-17	A
HORSE004	NA	17.4	Horse	QLD	Currumbin_Valley	8/22/11	HORSE004/Horse/QLD/Currumbin_Valley/2011-08-22	D
HORSE005	NA	17.9	Horse	NSW	Newrybar	8/15/11	HORSE005/Horse/NSW/Newrybar/2011-08-15	U
HORSE006	NA	17.9	Horse	NSW	Mullumbimby	7/26/11	HORSE006/Horse/NSW/Mullumbimby/2011-07-26	U
HORSE007	NA	18.7	Horse	NSW	Pimlico	8/15/11	HORSE007/Horse/NSW/Pimlico/2011-08-15	D
HORSE008	NA	20.8	Horse	NSW	Tintenbar	8/28/11	HORSE008/Horse/NSW/Tintenbar/2011-08-28	U
HORSE009	NA	14.5	Horse	NSW	South_Arm	6/6/13	HORSE009/Horse/NSW/South_Arm/2013-06-06	C
JN255800	NA	NA	Bat	QLD	Yeppoon	8/15/09	JN255800/Bat/QLD/Yeppoon/2009-08-15	D
JN255801	NA	NA	Bat	QLD	Yeppoon	8/13/09	JN255801/Bat/QLD/Yeppoon/2009-08-13	D
JN255802	NA	NA	Bat	QLD	Tolga	8/24/09	JN255802/Bat/QLD/Tolga/2009-08-24	D
JN255803	NA	NA	Bat	QLD	Cedar_Grove	8/5/09	JN255803/Bat/QLD/Cedar_Grove/2009-08-05	D
JN255804	NA	NA	Horse	QLD	Clifton_Beach	7/18/07	JN255804/Horse/QLD/Clifton_Beach/2007-07-18	U
JN255805	NA	NA	Human	QLD	Redlands	7/15/08	JN255805/Human/QLD/Redlands/2008-07-15	U
JN255806	NA	NA	Horse	QLD	Cawarral	7/28/09	JN255806/Horse/QLD/Cawarral/2009-07-28	A
KY425627	NA	NA	Human	QLD	Brisbane	9/27/94	KY425627/Human/QLD/Brisbane/1994-09-27	U
MN062017	NA	NA	Horse	QLD	Mackay	8/1/94	MN062017/Horse/QLD/Mackay/1994-08-01	U
NC_001906	NA	NA	Horse	QLD	Brisbane	9/25/94	NC_001906/Horse/QLD/Brisbane/1994-09-25	U
RSIM001_NB-U-25	100.0	30	Bat	NSW	Simpsons_Creek	8/23/17	RSIM001_NB_U_25/Bat/NSW/Simpsons_Creek/2017-08-23	C
PQ660692	NA	NA	Horse	NSW	Cardiff_Heights	7/15/23	PQ660692/Horse/NSW/Cardiff_Heights/2023-07-15	B

*Sampled four individual bats: ACCLU001_AVL_U_16_1 (subadult, male), ACTOW001_AVL_U_13_1 (adult, female), ACTOW001_AVL_U_26_1 (adult, female), and ACTOW013_VTM_U_34_2 (adult, male). All sampled bats were black flying foxes

G gene sequences recovered by LRPCR only

strain		Ct	Species	State	Site	Date (mm/dd/yy)	Label	Clade
ARCLU007_AVL_U_63_1	NA	23	Bat	NSW	Clunes	8/1/18	ARCLU007_AVL_U_63_1/Bat/NSW/Clunes/2018-08-01	B
ARCLU008_AVL_U_11_1	NA	33	Bat	NSW	Clunes	8/30/18	ARCLU008_AVL_U_11_1/Bat/NSW/Clunes/2018-08-30	A
ARLIS002_AVL_U_22_1	NA	33	Bat	NSW	Lismore	8/27/17	ARLIS002_AVL_U_22_1/Bat/NSW/Lismore/2017-08-27	U
ARCAN002_AVL_U_29_1	NA	31	Bat	QLD	Canungra	8/28/17	ARCAN002_AVL_U_29_1/Bat/QLD/Canungra/2017-08-28	C
ARSIM001_AVL_U_33_1	NA	29	Bat	NWS	Simpson's Creek	8/23/17	ARSIM001_AVL_U_33_1/Bat/NWS/Simpson's Creek/2017-08-23	D
ARMUL001_AVL_U_1_1_2	NA	28	Bat	NSW	Mullumbimby	9/5/17	ARMUL001_AVL_U_1_1_2/Bat/NSW/Mullumbimby/2017-09-05	C
ARCUR001_AVL_U_21_1	NA	34	Bat	QLD	Currumbin_Valley	10/4/18	ARCUR001_AVL_U_21_1/Bat/QLD/Currumbin_Valley/2018-10-04	A
ARNAM002_AVL_U_42_1	NA	27	Bat	NSW	Nambuca_Heads	9/2/17	ARNAM002_AVL_U_42_1/Bat/NSW/Nambuca_Heads/2017-09-02	B
RCLU001_NB_U_05_023	NA	34	Bat	NSW	Clunes	7/17/17	RCLU001_NB_U_05_023/Bat/NSW/Clunes/2017-07-17	A
RCLU003_NB_U_16	NA	36	Bat	NSW	Clunes	9/4/17	RCLU003_NB_U_16/Bat/NSW/Clunes/2017-09-04	A
ARRED001_AVL_U_19_1	NA	33	Bat	QLD	Redcliffe	4/27/18	ARRED001_AVL_U_19_1/Bat/QLD/Redcliffe/2018-04-27	D
ARRED005_AVL_U_23	NA	32	Bat	QLD	Redcliffe	8/28/18	ARRED005_AVL_U_23/Bat/QLD/Redcliffe/2018-08-28	D
ARSUN014_AVL_U_9_1	NA	28	Bat	QLD	Sunnybank	10/27/18	ARSUN014_AVL_U_9_1/Bat/QLD/Sunnybank/2018-10-27	D
JN255813	NA	NA	Horse	QLD	Peachester	6/1/07	JN255813/Horse/QLD/Peachester/2007-06-01	U
JN255817	NA	NA	Human	QLD	Redlands	7/1/08	JN255817/Human/QLD/Redlands/2008-07-01	U
JN255815	NA	NA	Human	QLD	Redlands	7/1/08	JN255815/Human/QLD/Redlands/2008-07-01	U
JN255814	NA	NA	Bat	QLD	Gordonvale	11/20/08	JN255814/Bat/QLD/Gordonvale/2008-11-20	U
JN255812	NA	NA	Horse	QLD	Bowen	9/1/09	JN255812/Horse/QLD/Bowen/2009-09-01	U
JN255818	NA	NA	Human	QLD	Cawarral	8/1/09	JN255818/Human/QLD/Cawarral/2009-08-01	A
