# Supplementary material for: Spatio-temporal dynamics of Hendra virus in Australia reveal stable maintenance of diverse viral clades among Pteropus bats
Source: Nat Microbiol. 2026 Apr 7;11(4):851–66. doi: 10.1038/s41564-025-02254-7 (PMC13056563; doi:10.1038/s41564-025-02254-7)
Supplement: Supplementary file 5 — Cytochrome c oxidase subunit 1 predicted host species and accessions of all sequences generated in this study [file 41564_2025_2254_MOESM5_ESM.pdf]

## Supplementary Data 2 | Cytochrome c oxidase subunit 1 predicted host species and accessions of all sequences generated in this study

| Sample | GenBank accession | Species observed | Species COX1 sequence | Top species read pair | Total read pair classified | Percent top species of read pair |
|---|---|---|---|---|---|---|
| ACCLU001_AVL_U_16_1 | PV730172 | bff | *P. alecto* | 254.5 | 337.5 | 75.4 |
| ACTOW001_AVL_U_13_1 | PV730173 | bff | *P. alecto* | 203 | 203 | 100 |
| ACTOW001_AVL_U_26_1 | PV730174 | bff | *P. alecto* | 198.5 | 293.5 | 67.6 |
| ACTOW013_VTM_U_34_2 | PV730175 | bff | *P. alecto* | 455.5 | 455.5 | 100 |
| ARCAN002_AVL_U_33_1 | PV730176 | bff-gff | *P. alecto* | 946 | 1739.5 | 54.4 |
| ARCLU001_AVL_U_023_A_1 | PV730177 | bff | *P. alecto* | 4252 | 5631.5 | 75.5 |
| ARCLU002_AVL_U_7_1 | PV730178 | bff | *P. alecto* | 229.5 | 474 | 48.4 |
| ARCLU003_AVL_U_1_1 | PV730179 | bff | *P. alecto* | 24 | 24 | 100 |
| ARCLU003_AVL_U_16_1 | PV730180 | bff | *P. alecto* | 1995 | 2979.5 | 67 |
| ARCLU007_AVL_U_11_1 | PV730181 | bff-gff | *P. alecto* | 178 | 354.5 | 50.2 |
| ARCLU007_AVL_U_43_1 | PV730182 | Bat | *P. alecto* | 3325.5 | 5270.5 | 63.1 |
| ARCLU008_AVL_U_17_1 | PV730183 | bff | *P. alecto* | 4826 | 6004.5 | 80.4 |
| ARCLU015_VTM_U_41_1 | PV730184 | bff | *P. alecto* | 85.5 | 164.5 | 52 |
| ARCLU023_VTM_U_56_1 | PV730185 | bff-gff | *P. alecto* | 18 | 18 | 100 |
| ARCLU025_VTM_U_32_1 | PV730186 | bff-gff | *P. alecto* | 9.5 | 13 | 73.1 |
| ARCLU029_VTM_U_41_1 | PV730187 | gff | NP | NR | NR | NP |
| ARCUR001_AVL_U_3_1 | PV730188 | Bat | *P. alecto* | 731.5 | 1111.5 | 65.8 |
| ARHVB001_AVL_U_11_1 | PV730189 | bff-gff | *P. alecto* | 1320.5 | 1711.5 | 77.2 |
| ARLIS002_AVL_U_36_1 | PV730190 | bff | *P. alecto* | 297 | 490 | 60.6 |
| ARLIS002_AVL_U_50_1 | PV730191 | bff | *P. alecto* | 345.5 | 718.5 | 48.1 |
| ARMOM001_AVL_U_21_1 | PV730192 | bff-gff | *P. alecto* | 518.5 | 1260 | 41.2 |
| ARNAM001_AVL_U_21_1 | PV730193 | bff | NP | NR | NR | NP |
| ARNAM002_AVL_U_46_1 | PV730194 | Bat | *P. alecto* | 91 | 479 | 19 |
| ARNAM006_AVL_U_14_1 | PV730195 | bff | *P. alecto* | 19 | 105 | 18.1 |
| ARRED004_AVL_U_23_1 | PV730196 | bff-gff | *P. alecto* | 1486 | 2599.5 | 57.2 |
| ARRED005_AVL_U_14_1 | PV730197 | bff | NP | NR | NR | NP |
| ARRED007_AVL_U_49_1 | PV730198 | bff | *P. alecto* | 1806 | 2188.5 | 82.5 |
| ARRED028_VTM_U_4_1 | PV730199 | bff | *P. alecto* | 1233.5 | 1568 | 78.7 |
| ARRED030_VTM_U_8_1 | PV730200 | bff | *P. alecto* | 534 | 722 | 74 |
| ARSIM001_AVL_U_25_1 | PV730201 | bff-gff | *P. alecto* | 10691 | 12521.5 | 85.4 |
| ARSUN014_AVL_U_4_1 | PV730202 | bff | *P. alecto* | 6486 | 7601 | 85.3 |
| ARSUN036_VTM_U_20_1 | PV730203 | bff | NP | NR | NR | NP |
| ARSUN038_VTM_U_48_1 | PV730204 | gff | NP | NR | NR | NP |
| ARTOW002_AVL_U_54_1 | PV730205 | bff | *P. alecto* | 124.5 | 146.5 | 85 |

| | | | | | | |
|---|---|---|---|---|---|---|
| *ARTOW003_VTM_U_52_1 | PV730206 | bff | *C. sabaeus* | 61 | 265.5 | 23 |
| ARTOW004_AVL_U_3_1 | PV730207 | bff | *P. alecto* | 1157.5 | 1972.5 | 58.7 |
| ARTOW004_AVL_U_38_1 | PV730208 | bff | *P. alecto* | 2438.5 | 2676.5 | 91.1 |
| ARTOW004_AVL_U_39_1 | PV730209 | bff | *P. alecto* | 852.5 | 1430 | 59.6 |
| ARTOW004_AVL_U_4_1 | PV730210 | bff | NP | NR | NR | NP |
| ARTOW005_AVL_U_16_1 | PV730211 | bat | *P. alecto* | 111 | 190 | 58.4 |
| ARTOW006_AVL_U_10_1 | PV730212 | bff | *P. alecto* | 66 | 66 | 100 |
| ARTOW006_AVL_U_14_1 | PV730213 | bff | *P. alecto* | 46 | 196.5 | 23.4 |
| ARTOW006_AVL_U_6_1 | PV730214 | bff | *P. alecto* | 100 | 156 | 64.1 |
| *ARTOW006_VTM_U_14_1 | PV730215 | bff | *C. sabaeus* | 890 | 1274 | 69.9 |
| ARTOW007_AVL_U_11_1 | PV730216 | bff-gff | *P. alecto* | 482 | 1128.5 | 42.7 |
| ARTOW022_VTM_U_51_1 | PV730217 | bff | NP | NR | NR | NP |
| ARTOW023_VTM_U_21_1 | PV730218 | bff | *P. alecto* | 890 | 906.5 | 98.2 |
| HORSE001 | PV730219 | Horse | NA | NA | NA | NA |
| HORSE002 | PV730220 | Horse | NA | NA | NA | NA |
| HORSE003 | PV730221 | Horse | NA | NA | NA | NA |
| HORSE004 | PV730222 | Horse | NA | NA | NA | NA |
| HORSE005 | PV730223 | Horse | NA | NA | NA | NA |
| HORSE006 | PV730224 | Horse | NA | NA | NA | NA |
| HORSE007 | PV730225 | Horse | NA | NA | NA | NA |
| HORSE008 | PV730226 | Horse | NA | NA | NA | NA |
| HORSE009 | PV730227 | Horse | NA | NA | NA | NA |
| RSIM001_NB_U_25 | PV730228 | bff-gff | *P. alecto* | 169 | 460 | 36.7 |

NR = No COX1 reads, bff = black flying fox, gff = grey flying fox, COX1 = Cytochrome c oxidase subunit I, *P. alecto* = *Pteropus alecto* , *Chlorocebus sabaeus* = *C. sabaeus,* * = sequence was obtained from cell culture isolate (isolated on Vero cells (African green monkey), NA = not applicable.
