# Supplementary material for: Spatio-temporal dynamics of Hendra virus in Australia reveal stable maintenance of diverse viral clades among Pteropus bats
Source: Nat Microbiol. 2026 Apr 7;11(4):851–66. doi: 10.1038/s41564-025-02254-7 (PMC13056563; doi:10.1038/s41564-025-02254-7)
Supplement: Supplementary file 9 — Unprocessed western blots. [file 41564_2025_2254_MOESM9_ESM.pdf]

PaKiT Replicates

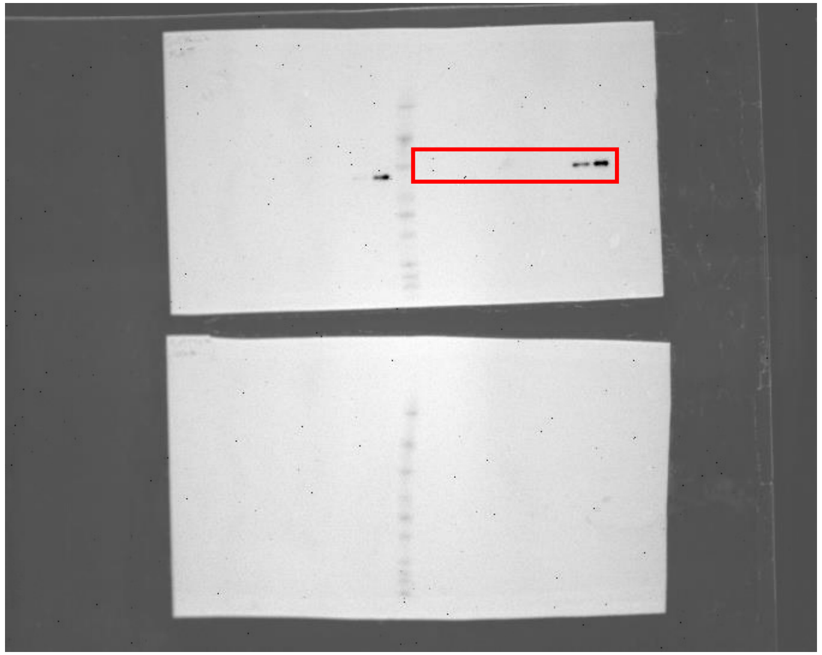

1. Rabbit anti-IFIT3, anti-rabbit HRP

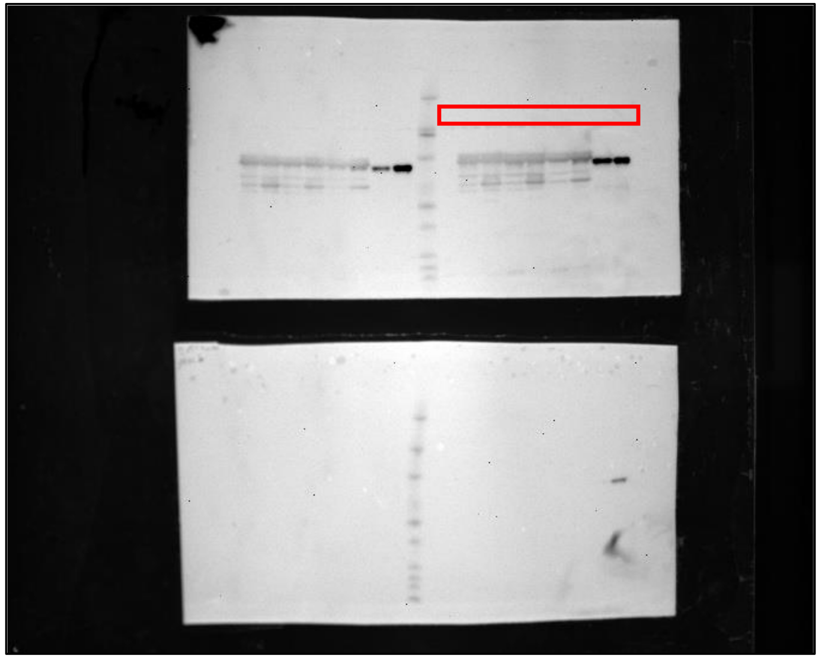

2. Rabbit anti-pSTAT2, anti-rabbit HRP

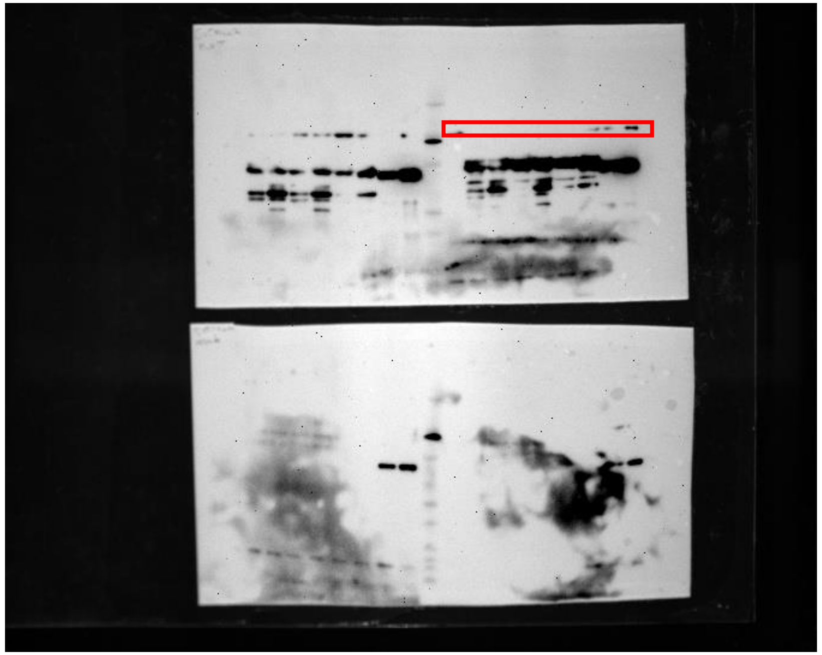

3. Rabbit anti-pSTAT1, anti-rabbit HRP

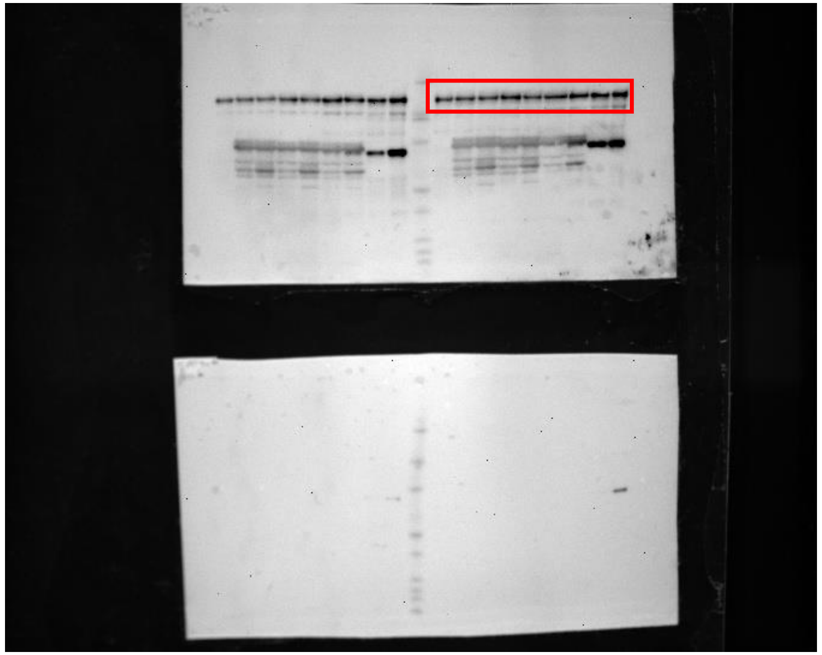

4. Rabbit anti-STAT2, anti-rabbit HRP

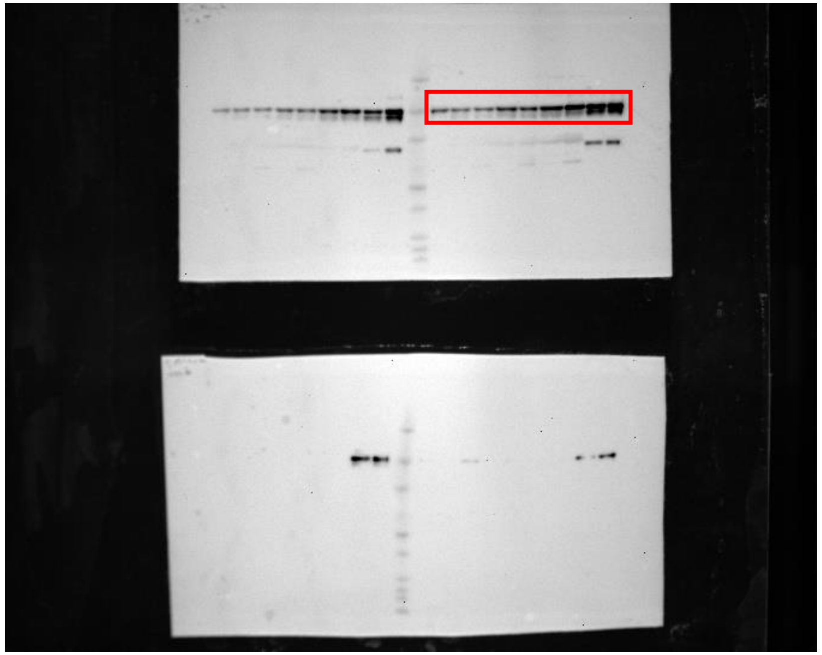

5. Rabbit anti-STAT1, anti-rabbit HRP

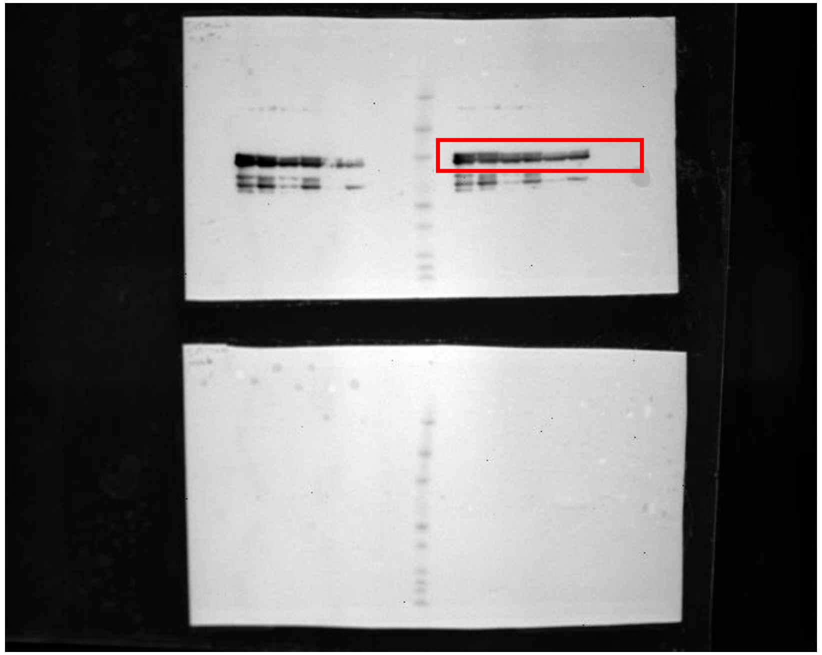

6. Rabbit anti-NiV N, anti-rabbit HRP

PaKiT Replicates

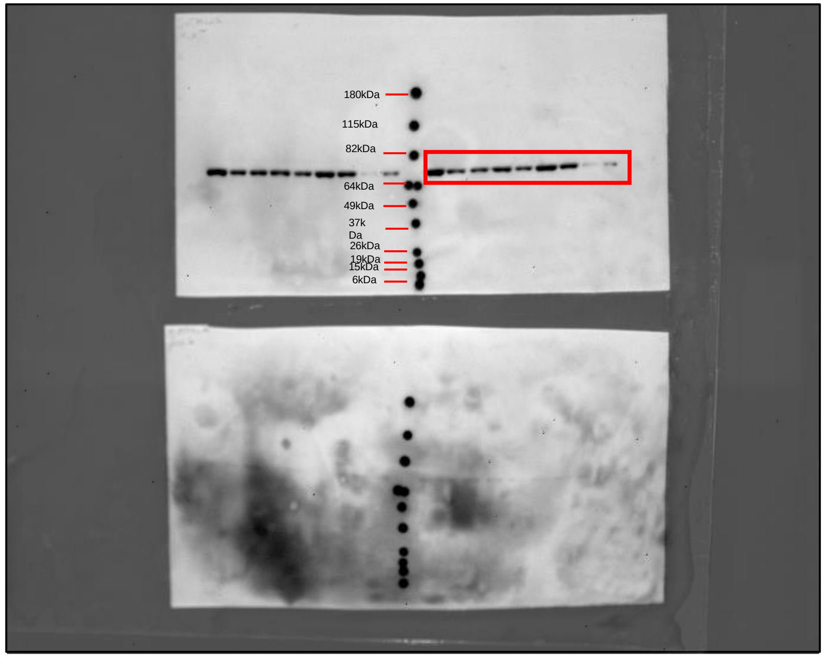

7. Mouse anti-beta tubulin, anti-mouse HRP

NBL-6 Replicates

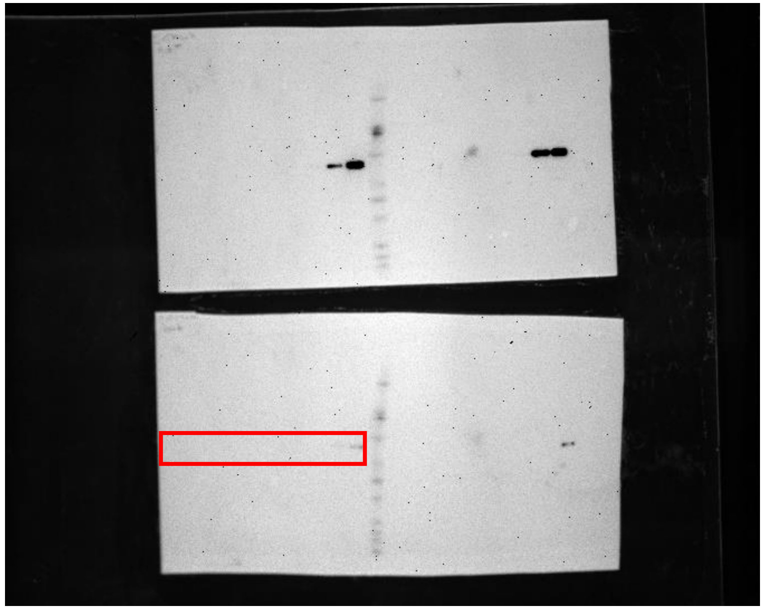

1. Rabbit anti-IFIT3, anti-rabbit HRP

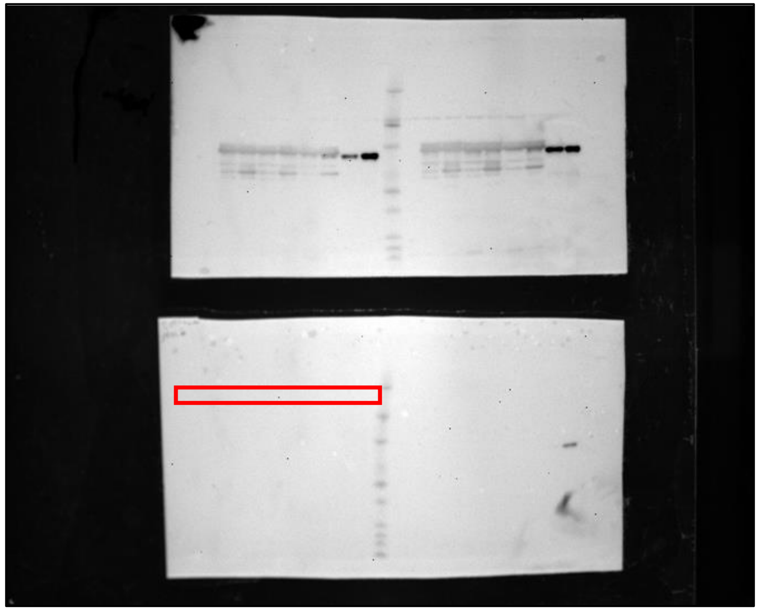

2. Rabbit anti-pSTAT2, anti-rabbit HRP

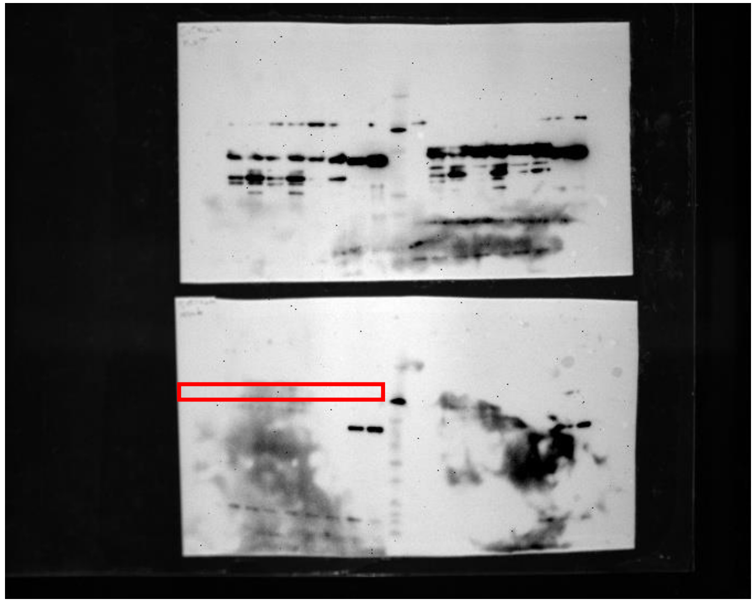

3. Rabbit anti-pSTAT1, anti-rabbit HRP

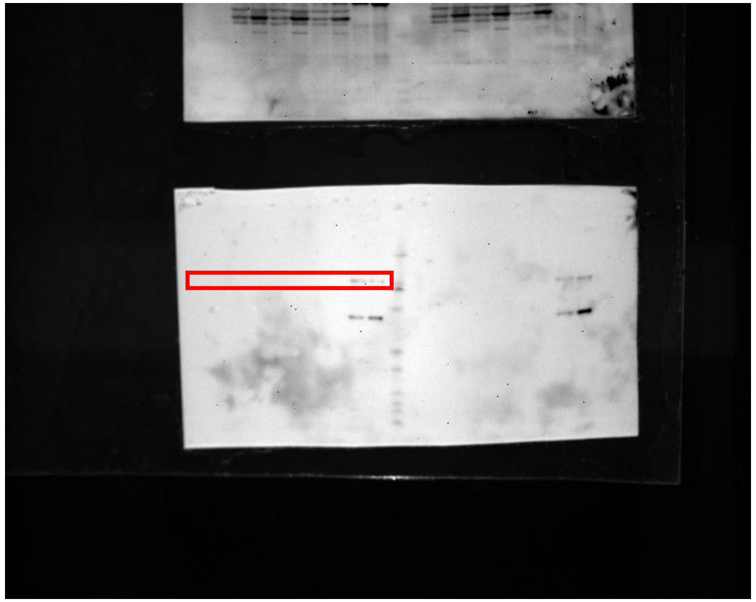

4. Rabbit anti-STAT2, anti-rabbit HRP

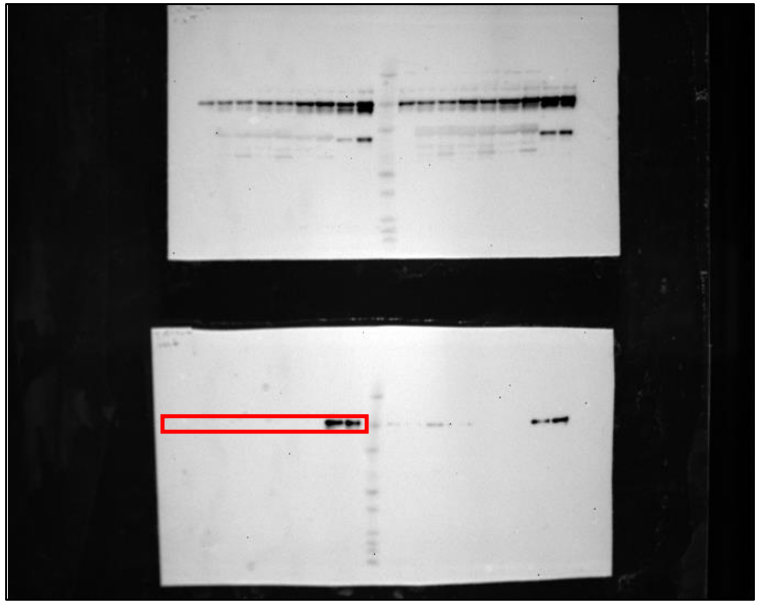

5. Rabbit anti-STAT1, anti-rabbit HRP

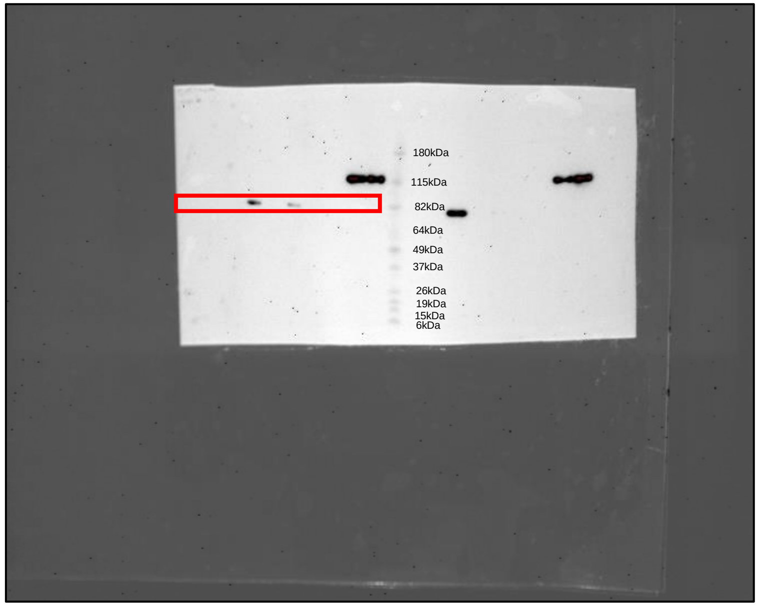

6. Rabbit anti-NiV N, anti-rabbit HRP

NBL-6 Replicates

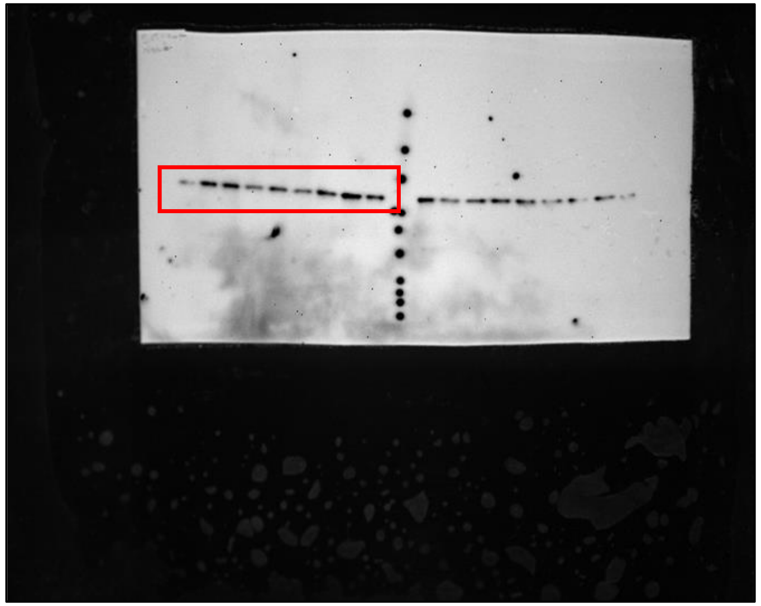

7. Mouse anti-beta tubulin, anti-mouse HRP

HFL-1 Replicates

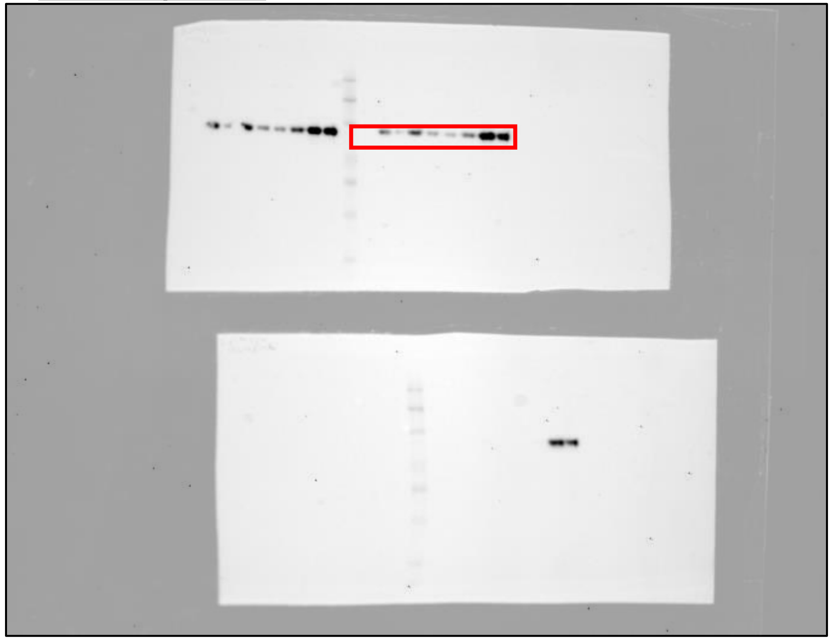

1. Rabbit anti-IFIT3, anti-rabbit HRP

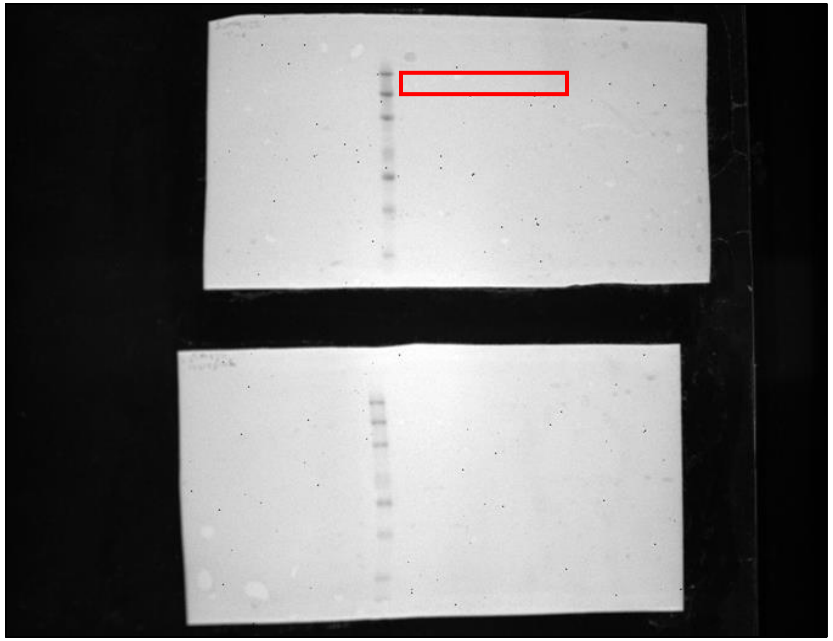

2. Rabbit anti-pSTAT2, anti-rabbit HRP

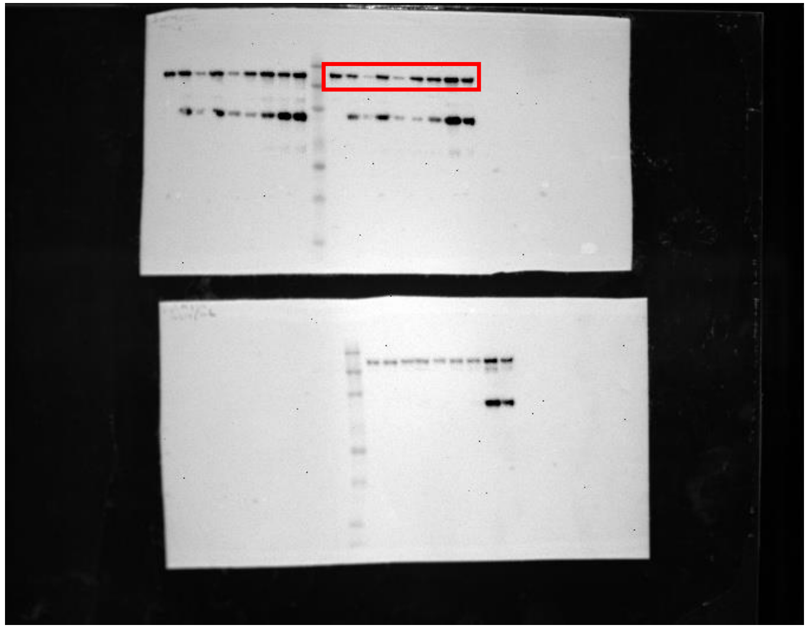

3. Rabbit anti-STAT2, anti-rabbit HRP

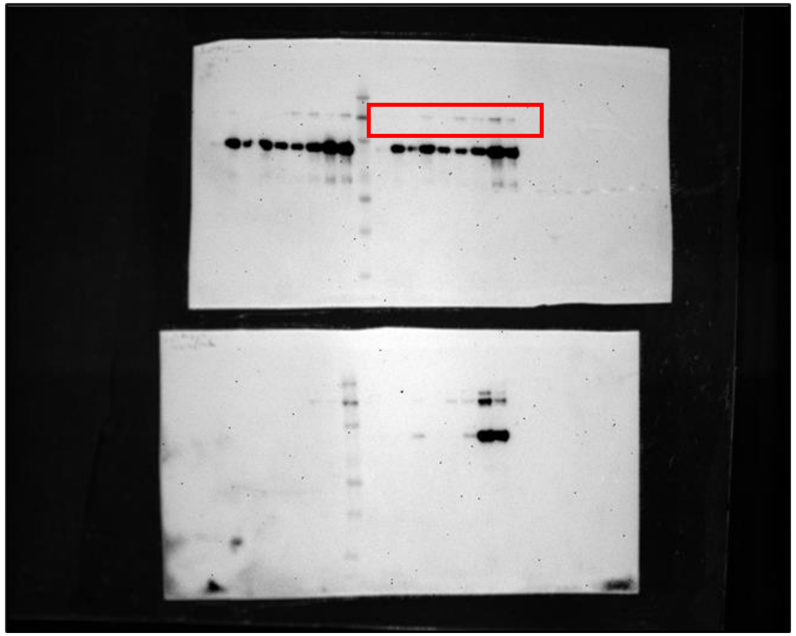

4. Rabbit anti-pSTAT1, anti-rabbit HRP

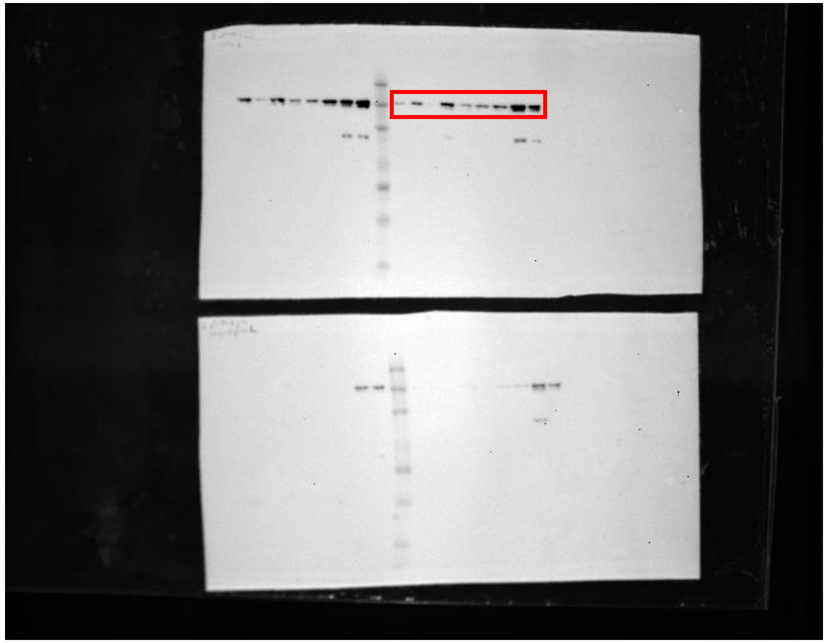

5. Rabbit anti-STAT1, anti-rabbit HRP

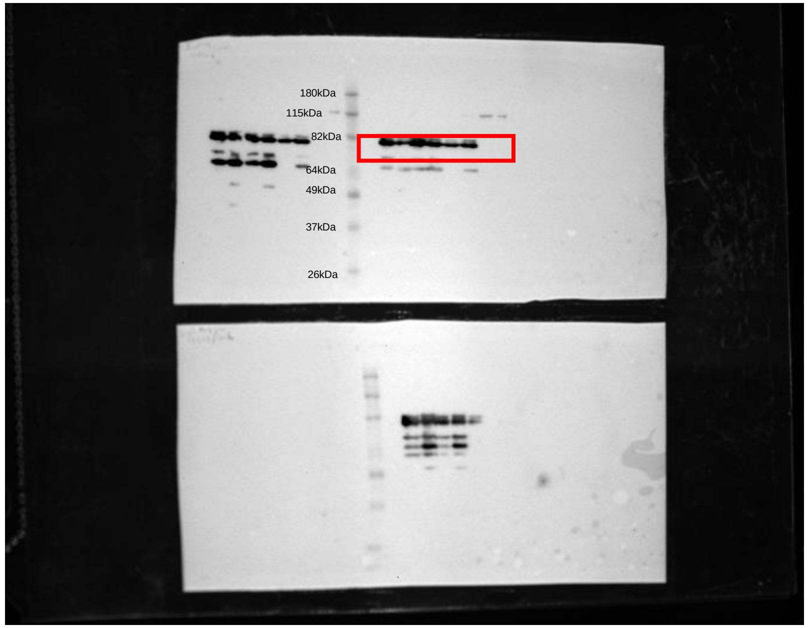

7. Rabbit anti-NiV N, anti-rabbit HRP

HFL-1 Replicates

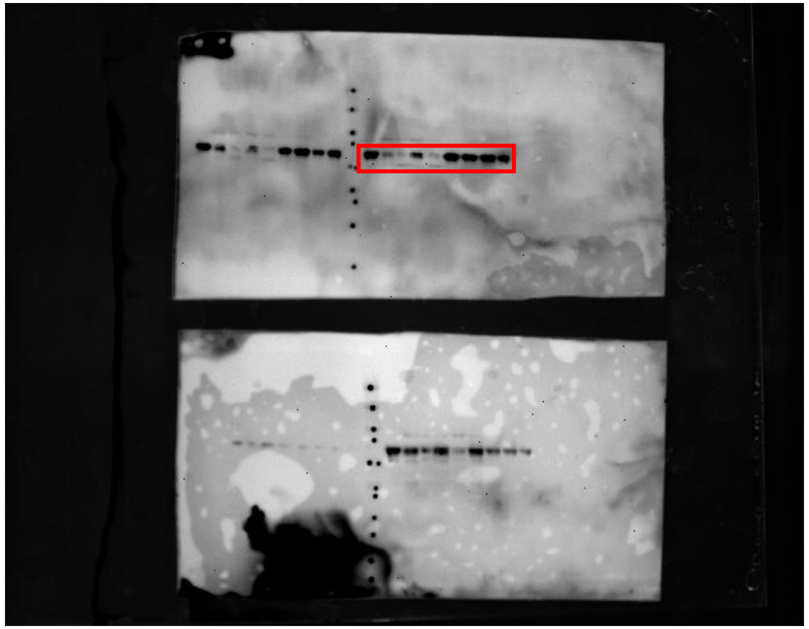

9. Mouse anti-beta tubulin, anti-mouse HRP
